# Identification of the *GST* Gene Family and Functional Analysis of *RcGSTF2* Related to Anthocyanin in *Rosa chinensis* ‘Old Blush’

**DOI:** 10.3390/plants14060932

**Published:** 2025-03-16

**Authors:** Ting Zhang, Han Wu, Yujia Sun, Peiheng Zhang, Lixia Li, Dan Luo, Zhe Wu

**Affiliations:** 1 College of Horticulture, Shanxi Agricultural University, Taigu 030801, China; zhang17891@163.com (T.Z.); wh01080709@163.com (H.W.); 17735736161@163.com (Y.S.); caoxiaoying2024@163.com (P.Z.); lilixia222@126.com (L.L.); danluo@sxau.edu.cn (D.L.); 2 Quzhou Academy of Agricultural and Forestry Sciences, Quzhou 324000, China

**Keywords:** rosa, *GST* genes, expression characteristics, *RcGSTF2*, anthocyanins

## Abstract

The rose (*Rosa chinensis*), with its rich color variations and elegant form, holds a significant position in the global floriculture industry, where the color of its petals and the content of anthocyanins are crucial for enhancing the plant’s ornamental value and market competitiveness. Nevertheless, the precise roles of the GST gene family in roses, especially regarding their participation in anthocyanin transport and the modulation of petal color, remain poorly elucidated. In the present investigation, we identified 83 rose glutathione S-transferase (GST) genes through whole-genome analysis. The identification and functional analysis of *RcGSTF2* were conducted exclusively in the ‘Old Blush’ cultivar of *Rosa chinensis*. We employed bioinformatics, tissue expression analysis, subcellular localization, and transient expression validation to explore the function of the *RcGSTF2* gene in anthocyanin transport and accumulation. We found that *RcGSTF2* is closely related to anthocyanin-associated GSTs and demonstrated a conserved domain with high sequence similarity. Molecular docking analysis revealed potential binding modes between RcGSTF2 and cyanidin-3,5-diglucoside, suggesting a role in anthocyanin transport. Subcellular localization indicated that *RcGSTF2* is associated with the cell membrane. Overexpression of *RcGSTF2* in rose plants significantly increased anthocyanin accumulation, while silencing *RcGSTF2* reduced anthocyanin content, highlighting its crucial role in regulating anthocyanin accumulation. This research investigates the functions of the GST gene family in roses, laying the groundwork for developing more colorful and resilient rose cultivars, with the functional analysis of *RcGSTF2* being a key contribution to the floriculture industry’s genetic enhancement efforts.

## 1. Introduction

Rose (*Rosa chinensis*), revered for its diverse color spectrum and graceful morphology, holds a significant position within the global horticultural and floricultural sectors [1]. The hue of its petals, with a particular emphasis on anthocyanin content, is paramount in ascertaining the ornamental value of roses and significantly influences their market competitiveness. Anthocyanins, being the primary determinants of petal color, also exert essential biological functions such as antioxidant and photoprotective activities, profoundly impacting the physiological and ecological characteristics of roses [2,3].

Glutathione S-transferase (GST) gene families are multifunctional enzymes that are essential for the growth and development of plants [4]. Their main functions include cellular detoxification through the catalysis of glutathione conjugation with electrophilic compounds, transforming toxic substances into less toxic or non-toxic forms to protect cells from damage [5]. Members of the *GST* gene family are also involved in plant hormone signaling, regulating plant growth and development, and antioxidant defense by scavenging reactive oxygen species and reactive nitrogen species, thereby protecting plant cells from oxidative damage and maintaining redox balance [6]. Certain members of the *GST* gene family also have molecular chaperone functions, aiding in the proper folding of proteins, preventing the aggregation of misfolded proteins, and protecting cells from stress-induced damage [7]. The *GST* gene family is also involved in regulating cell death pathways, playing a role in plant immune responses and programmed cell death [8]. Currently, the *GST* family has been identified in a variety of plants, including apple [9,10], cucumber [11], sweet potato [12], millet [13], rapeseed [14], and tomato [15]. These studies, by comparing the number, types, and expression patterns of *GST* genes in different plants, have revealed the diversity and functions of the *GST* gene family across various plant species. For instance, in apples, 38 *GST* genes were identified based on sequence similarity with Arabidopsis *GST* family proteins, which can be divided into nine major subclasses [10]. In cucumbers, 46 *GST* genes were identified, divided into 11 classes, and found to be involved in cold stress responses [11]. In sweet potato, 42 *GST* genes were identified, categorized into eight classes, and these genes exhibit differential responses to abiotic stresses in aboveground and underground tissues [12]. These studies provide valuable information for understanding the role of *GST* gene families in plants, yet research on roses remains scarce.

Furthermore, the *GST* gene family is involved in the transport and storage of secondary metabolites, particularly in the transport of pigment molecules like anthocyanins [16]. *GST* genes influence the color formation of petals and other tissues by transporting anthocyanins from the cytoplasm to the vacuoles, which has a significant impact on the ornamental value and nutritional value of fruits [17]. Research has identified specific *GST* genes in various species that are required for anthocyanin transport and accumulation, such as *An9* in petunia [18], *AtTT19* (*AtGSTF12*) in Arabidopsis [19], *FvRAP* in strawberry [20], *AcGST1* in kiwifruit [21], *MdGSTF6* in apple [22], *PcGST57* in pear [23], *PpGST1* in peach [24], *GhGSTF12* in cotton [25], and *LhGST* in Asiatic hybrid lilies [26]. Additionally, *PsGSTF3* in tree peony has been demonstrated to be a strong candidate for an anthocyanin transporter involved in the coloration of tree peony petals [27]. Although research on the *GST* gene family has made progress in various plants, research on roses is relatively limited, especially regarding the specific roles and regulatory mechanisms of *GST* genes in anthocyanin biosynthesis and color formation. This gap in knowledge limits our understanding of the mechanisms underlying petal color formation in roses and hinders the potential to improve the ornamental value of roses through genetic improvement.

In this study, we aim to identify members of the *GST* gene family in the *Rosa chinensis* ‘Old Blush’ cultivar and employ bioinformatics analysis, tissue expression analysis, subcellular localization, and transient expression validation to explore the function of the *RcGSTF2* gene in anthocyanin transport and accumulation. This research will deepen our understanding of the functions of the *GST* gene family in roses and provide a scientific basis for breeding new rose varieties with richer colors and stronger stress resistance by thoroughly analyzing the role of *GST* genes in anthocyanin metabolism, thereby promoting the continuous advancement and development of the floriculture industry.

## 2. Results

### 2.1. Identification and Characterization of GST Gene Family Members in Roses

In our study, we identified a total of 83 rose glutathione S-transferases (*RcGSTs*), which are listed in Tables S1 and S2. The protein lengths of these RcGSTs vary significantly, from the shortest RcGSTU6 with 99 amino acids to the longest RcEF1Bγ1 with 422 amino acids. The theoretical isoelectric points (pI) of the RcGSTs span a range, with RcGSTZ4 having the lowest pI of 4.94 and RcEF1Bγ3 the highest at 10.38. Similarly, the molecular weights of the RcGSTs extend from a low of 11.15 kDa for RcGSTU6 to a high of 47.71 kDa for RcEF1Bγ1.

**Figure 1:**
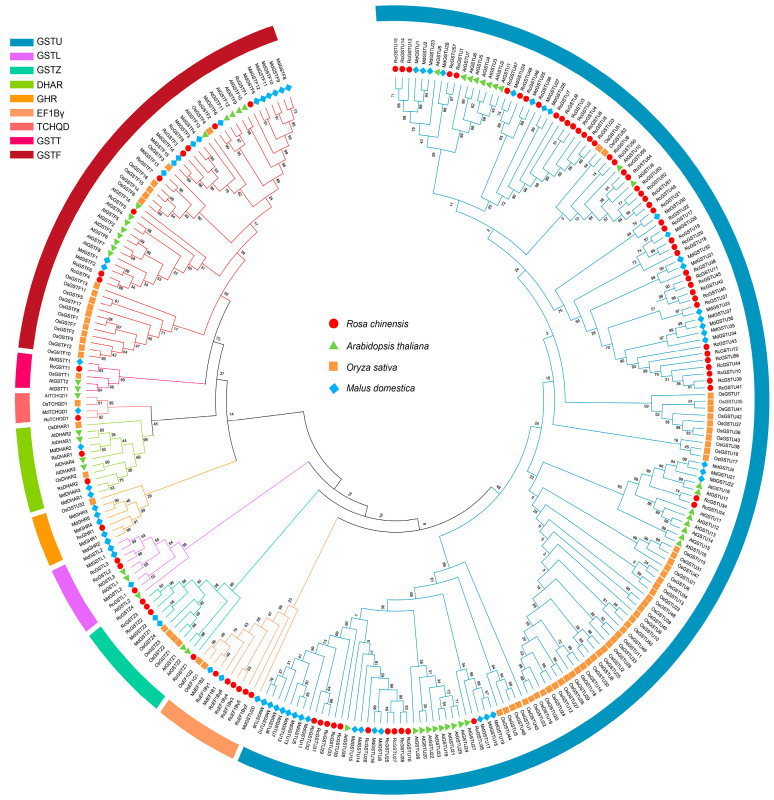
The phylogenetic tree of *GST* genes from rose, *Arabidopsis*, apple, and rice.

**Figure 2:**
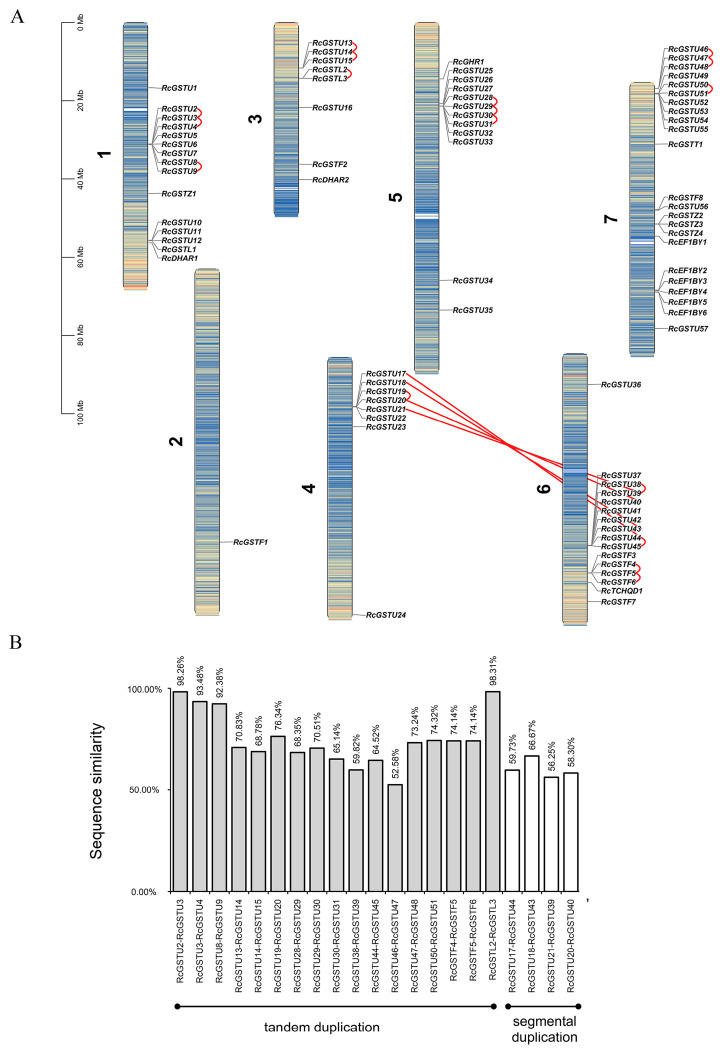
Genomic distribution and sequence similarity of *RcGSTs* in rose. (**A**) Chromosome distributions and synteny relationship of 83 *RcGSTs* in the rose genome. The heatmap represents gene density, with blue indicating low density and orange indicating high density. (**B**) Sequence similarity among the synteny gene pairs.

Phylogenetic tree construction revealed that GST proteins from rose, along with those from *Arabidopsis thaliana* (53 AtGSTs), *Malus domestica* (53 MdGSTs), and *Oryza sativa* (77 OsGSTs), are grouped into seven major subfamilies: Tau, Lambda, Phi, Zeta, TCHQD, DHAR, and Theta (Figure 1). Chromosome localization analysis indicated that the *RcGST* genes in roses are spread across seven distinct chromosomes, with chromosome 7 containing the highest number of GST genes, totaling 23, while chromosome 2 has the fewest with only one GST gene (Figure 2A).

**Figure 3:**
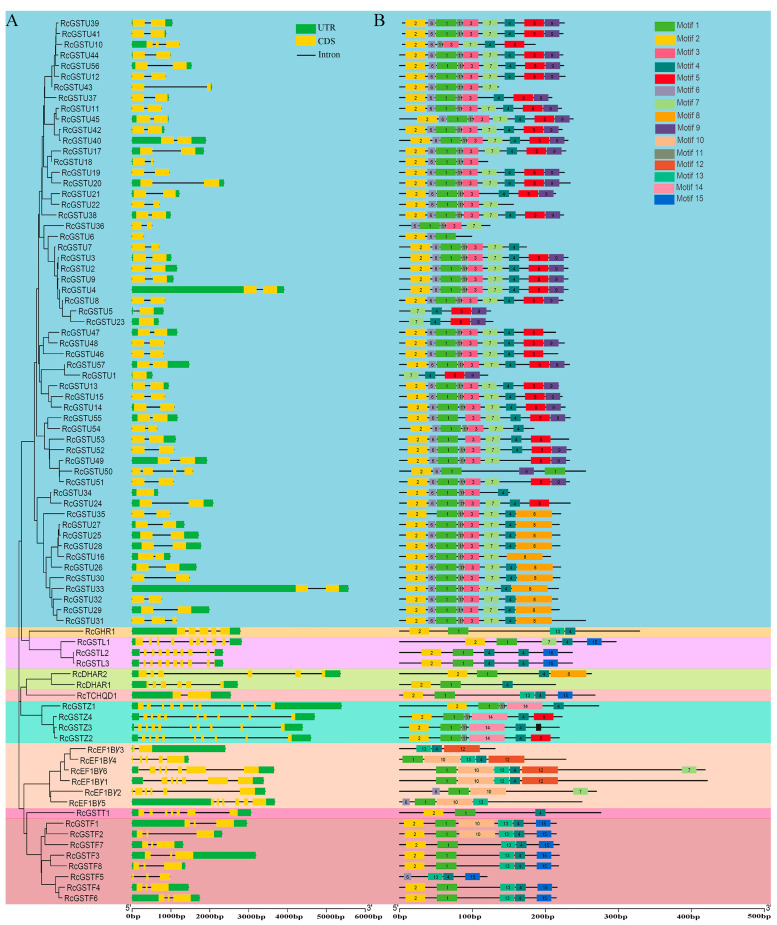
Structural and motif analysis of *RcGSTs* in rose. (**A**) Exon–intron organization of *RcGST* genes. (**B**) Composition and distributions of conserved motifs in RcGST proteins.

Investigation into gene family duplication within the rose genome revealed 21 pairs of duplicated *RcGST* genes (Figure 2B). Of these pairs, 17 were identified as tandem duplications and 4 as segmental duplications. The analysis of introns and exons in *RcGSTs* revealed significant structural diversity, with the number ranging from 1 to 10 (Figure 3A). Analysis of conserved motifs showed that the number of motifs per GST protein varies from one to six. The longest shared sequences are found in motif 12, which can include up to 50 amino acids, while the shortest shared sequence is in motif 11, containing only 8 amino acids (Table S3). Motif composition is consistent within certain subfamilies: the Phi subfamily consistently includes motifs 1, 2, 4, 13, and 15; the DHAR subfamily contains motifs 1, 2, and 4 (Figure 3B).

### 2.2. Functional Annotation of RcGSTs

**Figure 4:**
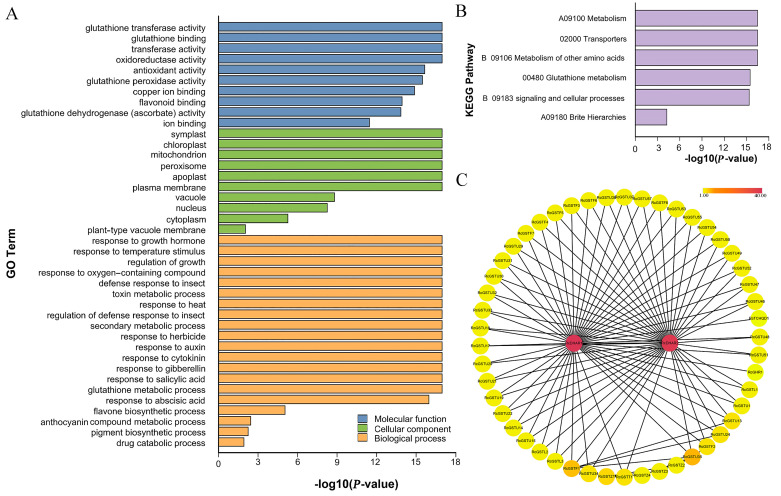
Functional annotation of RcGSTs. (**A**) GO function annotation of RcGST proteins. (**B**) KEGG enrichment of RcGST proteins. (**C**) Protein–protein interactions networks of RcGST members. The score was represented by a yellow–red gradient color: Yellow nodes indicate a low score, and red nodes indicate a high score.

Figure 4A displays the categorization of 83 *RcGST* genes into 40 Gene Ontology (GO) terms, encompassing 10 molecular functions (MF), 10 cellular components (CC), and 20 biological processes (BP). The RcGSTs were predominantly enriched in GO terms related to glutathione transferase activity, glutathione binding, transferase activity, and oxidoreductase activity within the MF category. In terms of cellular components, symplast, chloroplast, mitochondrion, peroxisome, and plasma membrane were significantly enriched. Additionally, the genes were primarily involved in responses to various hormones, including auxin, cytokinin, gibberellin, salicylic acid, and abscisic acid, as well as biotic (insect) and abiotic stresses (temperature stimulus, toxin, and herbicide) within the biological processes category. Notably, processes related to flavonol (GO:0051555) and anthocyanin (GO:0046283) metabolism were also enriched in BP.

Furthermore, KEGG enrichment analysis revealed that RcGST proteins are largely associated with metabolic pathways, transport functions, the metabolism of other amino acids, glutathione metabolism, signaling mechanisms, and various cellular processes (Figure 4B). These findings highlight the essential role of RcGST proteins in multiple key physiological processes within the cell.

Protein–protein interactions (PPIs), which regulate a wide array of cellular activities including the adjustment of metabolic pathways in plants [28], were investigated through an interaction network constructed with the STRING database. The PPI network analysis indicated that RcDHARs have a high degree of interaction with other RcGST members (Figure 4C). Among these, Tau members were the most numerous (31), followed by Phi members (8), Lambda members (3), Theta (1), GHR (1), and TCHQD (1), suggesting a complex network of interactions among the RcGST family members.

### 2.3. Expression Analysis of RcGST Transcripts in Different Tissues

**Figure 5:**
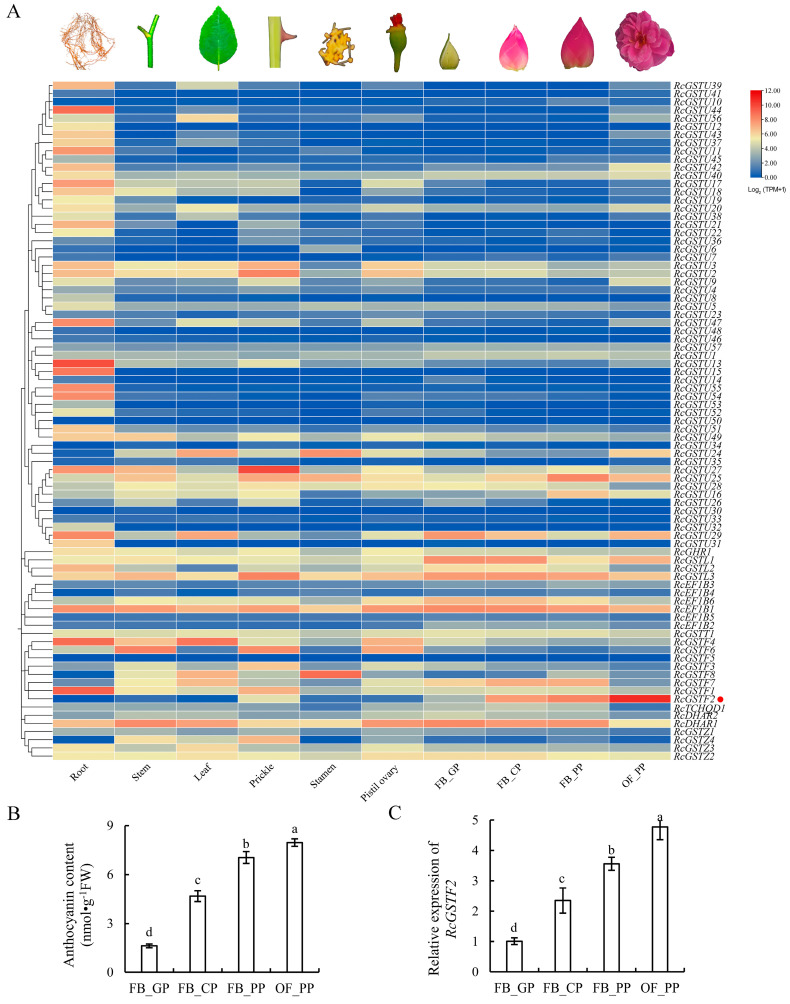
Comprehensive analysis of *RcGST* gene expression and anthocyanin content in rose petal development. (**A**) Expression patterns of *RcGST* genes in different tissues. Expression levels are shown as the Log2-transformed (TPM+1) values obtained from the RNA-Seq data. (**B**) The anthocyanin concentrations in the petals at four developmental stages: FB_GP, green petals in the flower bud; FB_CP, color-changing petals in the flower bud; FB_PP, pink petals in the flower bud; OF_PP, pink petals of the open flower. (**C**) The relative expression levels of the *RcGSTF2* gene at the four stages of petal development in roses. The *RcACTIN* gene was used as the internal control. In panels (**B**,**C**), error bars represent the standard deviation (SD) from three independent experiments, each comprising three technical replicates. Data labeled with different lowercase letters indicate significant differences at *p* < 0.05.

To thoroughly elucidate the expression characteristics of the *RcGST* genes in roses, we analyzed two published RNA-seq datasets that covered a variety of tissues in the ‘Old Blush’ rose (Figure 5A, Table S4), including roots, stems, leaves, stamens, thorns, pistils, and ovaries [29], as well as transcriptome data during the development of petals in the ‘Old Blush’ rose, which were divided into four typical stages: green petals in young flower buds (FB_GP), color-changing petals in flower buds (FB_CP), pink petals in flower buds (FB_PP), and pink petals in open flowers (OF_PP) [30]. In various tissues of the rose, we observed that certain *RcGST* genes exhibit low expression levels or are hardly detectable, especially during petal development, such as *RcGSTU50*, *RcGSTU48*, *RcEF1B4*, and *RcEF1B5*. In contrast, other *RcGST* genes show relatively high expression levels across all tested tissues, like *RcGSTT1*, *RcEF1B1*, *RcGSTU28*, and *RcGSTU25*. Moreover, some *RcGST* genes display particularly high expression in specific tissues, such as *RcGSTF1*, *RcGSTU12*, *RcGSTU13*, *RcGSTU44*, and *RcGSTU53* in roots; *RcGSTU2* in thorns; *RcGSTF4* in both roots and leaves; and *RcGSTF8* in stamens. These expression patterns reveal the diversity and specificity of *RcGST* genes in the development and function of different rose tissues.

The expression pattern of *RcGSTF2* is particularly noteworthy. Its expression level in thorns is higher than that in roots, stems, leaves, stamens, pistils, and ovaries, which correlates with the phenotype of thorns containing a small number of anthocyanins and exhibiting a red color. Furthermore, *RcGSTF2* has a higher expression level in petals than in other tissues, and its expression increases gradually during petal development, which is consistent with the increasing trend of anthocyanin content during this process (Figure 5B). To further validate this, we used quantitative real-time PCR (qRT-PCR) to confirm that the expression trend of *RcGSTF2* during petal development is in agreement with the transcriptome data (Figure 5C). To further support the selection of RcGSTF2 as a candidate gene, we performed a correlation analysis between anthocyanin content and *RcGSTF2* expression levels across different flowering stages. The results showed a significant positive correlation (Pearson’s *r* = 0.96, *p* < 0.01), indicating that *RcGSTF2* expression is closely associated with anthocyanin accumulation. This analysis strengthens the rationale for focusing on *RcGSTF2* in our study.

### 2.4. Characterization of RcGSTF2 Gene Related to Anthocyanin Accumulation in Rose

**Figure 6:**
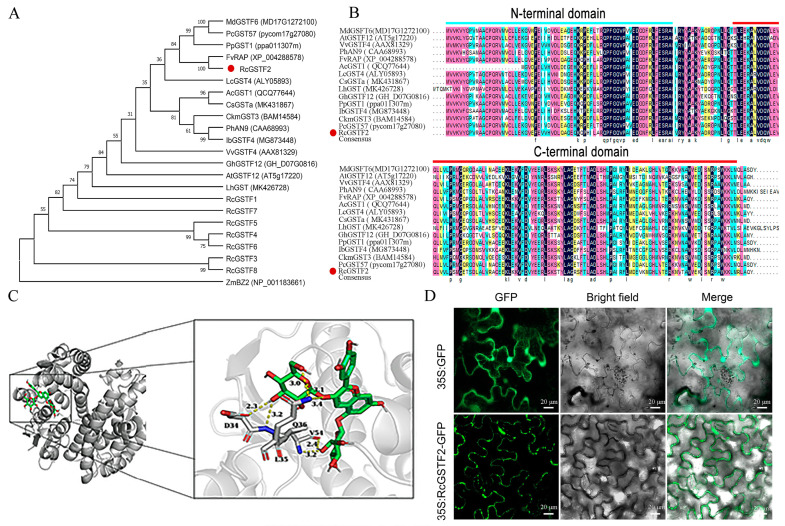
Characterization of *RcGSTF2* gene related to anthocyanin accumulation in rose. (**A**) Phylogenetic analysis of RcGSTF2 amino acid sequence with other anthocyanin-related GST sequences. MdGSTF6 (*Malus domestica*, MD17G1272100); PcGST57 (*Pyrus communis*, pycom17g27080); PpGST1 (*Prunus persica*, ppa011307m); FvRAP (*Fragaria vesca*, XP_004288578); LcGST4 (*Litchi chinensis*, ALY05893); AcGST1 (*Actinidia chinensis*, QCQ77644); CsGSTa (*Camellia sinensis*, MK431867); CkmGST3 (*Cyclamen*, BAM14584); PhAN9 (*Petunia hybrida*, CAA68993); IbGSTF4 (*Ipomoea batatas*, MG873448); VvGSTF4 (*Vitis vinifera*, AAX81329); GhGSTF12 (*Gossypium hirsutum*, GH_D07G0816); AtGSTF12 (*Arabidopsis thaliana*, AT5g17220); LhGST (*Lilium*, MK426728); ZmBZ2 (*Zea mays*, NP_001183661); (**B**) multiple sequence alignment of RcGSTF2 amino acids with anthocyanin-related GSTF genes; (**C**) molecular docking analysis of RcGSTF2 with cyanidin-3,5-diglucoside; (**D**) subcellular localization of RcGSTF2-GFP fusion protein in tobacco leaves.

We conducted a multiple sequence alignment and phylogenetic analysis of *RcGSTF2* with other known anthocyanin-related *GST* genes, as depicted in Figure 6A. The analysis revealed that RcGSTF2 is closely related to a range of anthocyanin-associated GSTs, such as *PhAN9* in petunia [18], *AtGSTF12* in Arabidopsis [19], *FvRAP* in strawberry [20], and *AcGST1* in kiwifruit [21]. Notably, sequence alignment demonstrated that RcGSTF2 shares a conserved domain and exhibits a high degree of sequence similarity, averaging 79.10%, with these anthocyanin-related GSTF genes (Figure 6B).

Building on these findings, we proceeded to investigate the interaction between the RcGSTF2 protein and cyanidin-3,5-diglucoside (Cy3G5G) through molecular docking analysis using AutoDock Vina software (version 1.2.x), with subsequent visualization using PyMOL software (version 3.1.1) (Figure 6C). The docking results exposed potential binding modes between Cy3G5G and the active site of RcGSTF2, highlighting the highest-scoring conformation that featured multiple hydrogen bonds between Cy3G5G and key amino acid residues of RcGSTF2. Moreover, the van der Waals interactions between Cy3G5G’s sugar moiety and RcGSTF2’s hydrophobic pocket were identified as crucial for the stability and specificity of the binding.

To elucidate the subcellular localization of RcGSTF2, we created a fusion protein by combining its coding sequence with the green fluorescent protein (GFP) (Figure 6D). The transient expression of this fusion protein in tobacco leaves resulted in a distinct fluorescence signal localized to the cell membrane, strongly indicating that RcGSTF2 is primarily associated with the cell membrane and likely plays a pivotal role in the regulation of anthocyanin transport.

### 2.5. Regulatory Role of RcGSTF2 in Anthocyanin Accumulation

**Figure 7:**
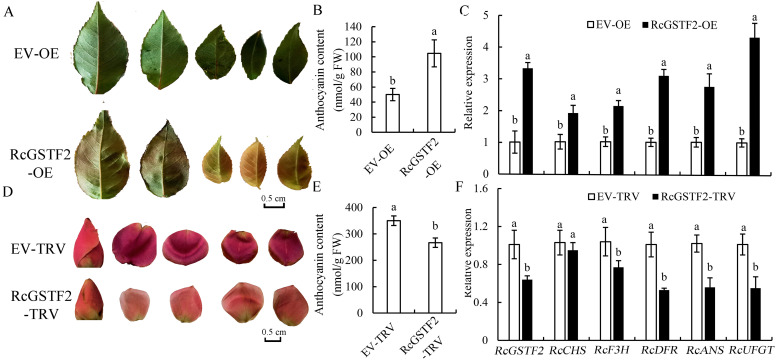
*RcGSTF2* is involved in the regulation of anthocyanin accumulation. (**A**) Transient overexpression of *RcGSTF2* in rose leaves injected with plasmid mixtures (EV-OE: pNC-Cam2304-MCS35S; RcGSTF2-OE: pNC-Cam2304-35S:*RcGSTF2*-GFP). An empty vector (pNC-Cam2304-MCS35S) was used as a control; (**B**) increased anthocyanin content in *RcGSTF2*-overexpressing rose leaves; (**C**) relative transcript levels of *RcGSTF2* and anthocyanin-related genes in rose leaves expressing EV-OE or *RcGSTF2*-OE. (**D**) Phenotype of rose petals after silencing *RcGSTF2* (EV-TRV: TRV1+TRV2; RcGSTF2-TRV: TRV1+ RcGSTF2-TRV2). An empty TRV vector was used as a control. (**E**) Anthocyanin content in rose petals after silencing *RcGSTF2*. (**F**) Relative transcript levels of *RcGSTF2* and anthocyanin-related genes in rose petals expressing EV-TRV or RcGSTF2-TRV. The *RcACTIN* gene was used as the internal control. In panels (**B**,**C**,**E**,**F**) error bars represent the standard deviation (SD) from three independent experiments, each comprising three technical replicates. Data labeled with different lowercase letters indicate significant differences at *p* < 0.05.

To gain a deeper understanding of the role of *RcGSTF2* in anthocyanin accumulation, we conducted a series of experiments involving both overexpression and gene silencing in rose plants. Figure 7A shows that the control leaves (EV-OE) remained green, while the leaves injected with the *RcGSTF2* overexpression plasmid (*RcGSTF2*-OE) turned significantly red. This result indicates that the overexpression of *RcGSTF2* induces anthocyanin accumulation in leaves, leading to the red coloration. This finding was further supported by the anthocyanin content analysis shown in Figure 7B, where the anthocyanin content in *RcGSTF2*-overexpressing leaves was approximately doubled compared to the control, highlighting the significant positive regulatory role of *RcGSTF2* in anthocyanin synthesis.

In Figure 7C, we analyzed the relative expression levels of *RcGSTF2* and key genes involved in anthocyanin biosynthesis (including *RcCHS*, *RcF3H*, *RcDFR*, *RcANS*, and *RcUFGT)* using qRT-PCR. The results revealed that the expression of these genes was significantly upregulated in *RcGSTF2*-overexpressing leaves. Furthermore, our experiments of the heterologous overexpression of *RcGSTF2* also significantly promoted anthocyanin accumulation in both apple peels and calli (Figure S1).

To further elucidate the function of *RcGSTF2*, we employed virus-induced gene silencing (VIGS) to silence *RcGSTF2* in the ‘Old Blush’ variety. The results showed that silencing *RcGSTF2* led to significantly lightened petal color (Figure 7D) and a substantial reduction in anthocyanin content (Figure 7E). This indicates that *RcGSTF2* is crucial for anthocyanin accumulation in petals. qRT-PCR analysis also revealed that, except for *RcCHS*, the expression of *RcGSTF2*, *RcF3H*, *RcDFR*, *RcANS*, and *RcUFGT* was significantly downregulated after silencing *RcGSTF2*. This suggests that *RcGSTF2* may influence anthocyanin accumulation by regulating the expression of these anthocyanin biosynthesis-related genes.

## 3. Discussion

In this study, we identified 83 *GST* genes in rosa, a discovery that expands the known diversity of the *GST* gene family in plants and is consistent with previous identifications of 92 *GST* genes in teinturier grape [31] and 53 *GST* genes in *Arabidopsis thaliana* [32]. The variations in protein length, isoelectric points, and molecular weights of these genes suggest potential functional specialization, aligning with the multifaceted roles of *GSTs* in plant physiology, including detoxification and responses to abiotic and biotic stresses, as discussed by Dixon et al. [33]. Phylogenetic analysis categorized RcGST proteins into seven subfamilies, indicating the conservation of GST functions across angiosperms [34]. This conservation implies that the functions of these genes may be evolutionarily preserved, providing a foundation for comparative studies and functional predictions. The uneven distribution of *RcGST* genes across rose chromosomes may indicate chromosomal regions as hotspots for gene expansion, a phenomenon observed in other plants and hypothesized to contribute to the adaptation and diversification of gene families [35]. These results not only reveal the diversity and complexity of the *GST* gene family in roses but also emphasize their potential roles in plant adaptability and evolution, offering new perspectives for future functional studies and comparative genomics research.

Building on this foundation, we conducted a comprehensive functional annotation analysis of the *GST* gene family in *Rosa chinensis*, revealing the multifaceted roles of these genes in plant physiological processes. Notably, our findings show that *RcGST* genes are closely associated with biological processes such as response to plant hormones and biotic and abiotic stresses, as well as flavonoid and anthocyanin metabolism. These results resonate with the research by Dixon et al. [36], which emphasized the importance of GSTs in the flavonoid pathway. KEGG enrichment analysis further revealed the potential roles of RcGST proteins in metabolic pathways, transport functions, glutathione metabolism, signal transduction mechanisms, and cellular processes, consistent with the key roles of GSTs in cell protection and signal transduction [37]. Protein–protein interaction (PPI) network analysis through the STRING database unveiled a complex interaction network among RcGST family members, suggesting the existence of an intricate regulatory network within the GST gene family that affects plant responses to environmental changes, supported by Szklarczyk et al. (2019) in their STRING database update [38]. These findings not only confirm the multifunctionality of the GST gene family in roses but also provide new perspectives for further exploration of the potential applications of these genes in rose genetic improvement and the development of new varieties.

In our in-depth functional analysis of the *RcGSTF2* gene, we found it to be closely associated with the accumulation of anthocyanins. This discovery is consistent with previous studies that have shown that the *GST* gene family plays a role in the transport of secondary metabolites in plants, including anthocyanins [39]. The research by Mueller et al. (2000) [18] revealed the role of a *GST* gene (*An9*) in anthocyanin sequestration in petunias, and our results, showing high expression levels of *RcGSTF2* in thorns and petals correlated with anthocyanin accumulation, further support the potential role of *GSTs* in the regulation of plant pigmentation. Molecular docking analysis unveiled potential binding modes between RcGSTF2 and cyanidin-3,5-diglucoside, providing new insights into how RcGSTF2 might be involved in anthocyanin transport at the molecular level [40]. This specific interaction suggests a conserved mechanism for *GSTs* in the transport of these pigments. The subcellular localization of RcGSTF2 to the cell membrane further supports its function in the transport of anthocyanins across cellular compartments, echoing the research by Edwards et al. [35] on the role of *GSTs* in vacuolar transport. Therefore, our results indicate that RcGSTF2 acts as a transport protein, facilitating the transport of anthocyanins from the cytoplasm to the vacuole, thereby influencing anthocyanin accumulation in rose petals.

Our experiments have demonstrated that overexpression of *RcGSTF2* in rose or apple tissues significantly enhances anthocyanin accumulation, as evidenced by the red coloration of leaves and an approximately twofold increase in anthocyanin content compared to controls. Conversely, silencing *RcGSTF2* using virus-induced gene silencing technology leads to a marked reduction in anthocyanin accumulation, further highlighting the pivotal role of *RcGSTF2* in anthocyanin accumulation. The upregulation of *RcGSTF2* expression in transgenic rose tissues correlates with increased anthocyanin content, whereas silencing *RcGSTF2* results in a significant decrease in anthocyanin levels, providing robust evidence for the gene’s involvement in anthocyanin regulation. These findings are consistent with the known function of *GSTs* in the transport of secondary metabolites, including anthocyanins, and further validate the effectiveness of heterologous systems for characterizing gene functions across different species. Aligning with the extensive research on *GSTs* in anthocyanin metabolism, including comprehensive reviews of flavonoid biosynthesis [36], our results not only confirm the crucial role of *RcGSTF2* in anthocyanin biosynthesis but also underscore the significance of GST gene family members in plant metabolic pathways.

Our research has uncovered a novel dimension of *RcGSTF2*’s function, revealing its pivotal role in orchestrating anthocyanin accumulation through dual mechanisms of transport facilitation and biosynthetic regulation. By overexpressing *RcGSTF2* in rose tissues, we observed a significant upregulation of key anthocyanin biosynthetic genes—*RcCHS*, *RcF3H*, *RcDFR*, *RcANS*, and *RcUFGT*—culminating in elevated anthocyanin levels. This stands in stark contrast to the VIGS-mediated silencing of *RcGSTF2*, which resulted in diminished anthocyanin content and concomitant suppression of the same biosynthetic genes. Such dual-pronged regulation underscores *RcGSTF2*’s central role in both anthocyanin transport and biosynthesis, drawing parallels with the distinct functions of *MdGSTF6* and *MdGSTU12* in apple [10,22]. Notably, while *MdGSTF6* silencing exerted minimal influence on anthocyanin biosynthetic genes, *MdGSTU12* knockdown profoundly altered their expression, indicative of a more intricate regulatory network.

The significant correlation between *RcGSTF2* expression and anthocyanin accumulation is attributable to multiple mechanisms. *RcGSTF2*-mediated vacuolar sequestration of anthocyanins may relieve cytoplasmic feedback inhibition, thereby augmenting biosynthetic gene expression [36]. Moreover, *RcGSTF2* may interact with transcription factors or structural genes in the anthocyanin biosynthesis pathway. For example, in apples, MdGST12 interacts with MdUFGT and MdDFR, enhancing *MdDFR* promoter activation and complicating light-induced anthocyanin biosynthesis regulation [41]. Additionally, *RcGSTF2*’s involvement in intracellular signaling pathways, such as hormone signaling or stress responses, might indirectly influence gene transcription [4,5]. Collectively, these mechanisms underscore *RcGSTF2*’s role as a dynamic modulator of secondary metabolism, expanding the traditional paradigm of plant *GSTs* beyond their passive transport functions.

While this study has provided valuable insights, it is essential to acknowledge its limitations. The focus on a single rose variety may restrict the generalizability of the findings. Future research should aim to broaden the scope by examining the functional conservation of *RcGSTF2* across different rose varieties and under various environmental conditions, as suggested by studies on the plasticity of *GST* gene functions.

## 4. Materials and Methods

### 4.1. Data Source and RcGST Gene Identification

The genomic sequences and their corresponding annotations for the rose species (*Rosa chinensis* ‘Old Blush’) were sourced from the *Rosa chinensis* OldBlush Hm r2.0 genome portal, accessible at https://lipm-browsers.toulouse.inra.fr/pub/RchiOBHm-V2/ (accessed on 18 October 2024) [1]. Sequences of glutathione S-transferases (GSTs) from Arabidopsis, denoted as *AtGSTs*, and from rice, referred to as *OsGSTs*, were retrieved from the respective databases: The Arabidopsis Information Resource (TAIR) at https://www.arabidopsis.org/ (accessed on 18 October 2024) and the Rice Genome Annotation Project at http://rice.uga.edu/index.shtml (accessed on 18 October 2024). Additionally, sequences for apple, referred to as *MdGSTs*, were sourced from the Rosaceae Genome Database, which can be accessed at https://www.rosaceae.org/ (accessed on 18 October 2024). These sequences served as the basis for conducting a local BLASTP search against the rose genome’s protein database, aiming to identify all potential GST family members with an E-value threshold of ≤1 × 10^−10^ and a sequence identity of at least 50%. In addition, the Hidden Markov Model (HMM) profiles for the GST-N domain (PF02798) and GST-C domain (PF00043) were procured from the Pfam database (http://pfam.xfam.org/) (accessed on 23 October 2024) and employed to search for putative members of the RcGST family using HMMER 3.1, with an E-value cutoff of ≤1 × 10^−5^. After eliminating any redundant sequences, the remaining RcGST candidates were further authenticated by examining the presence of conserved domains through the use of the SMART tool, available at http://smart.embl-heidelberg.de/ (accessed on 23 October 2024), and the NCBI Conserved Domain Database (CDD), accessible at https://www.ncbi.nlm.nih.gov/Structure/cdd/wrpsb.cgi (accessed on 23 October 2024).

### 4.2. Phylogenetic Analysis and Subfamily Categorization

Alignment of GST protein sequences across Arabidopsis, apple, rice, and rose was conducted using Clustal Omega with default parameters, found at https://www.ebi.ac.uk/Tools/msa/clustalo/ (accessed on 23 October 2024). A phylogenetic tree was constructed using the neighbor-joining algorithm in MEGA X software, with 1000 bootstrap replicates (http://www.megasoftware.net/, accessed on 23 October 2024). Based on the subfamily classification in Arabidopsis, apple, and rice, the *RcGST* genes were sorted into various subfamilies. For a precise prediction of their molecular functions, a phylogenetic tree was constructed using *GST* genes related to anthocyanin accumulation along with *RcGST* genes, following the same procedures outlined above. The biochemical characteristics of the RcGST proteins, including polypeptide length, molecular weight, and isoelectric point, were analyzed using the ProtParam tool available at the ExPasy server, accessible at http://web.expasy.org/protparam/ (accessed on 23 October 2024). To forecast the subcellular distribution of the RcGSTs, three distinct online prediction tools were employed: CELLO v.2.5 at http://cello.life.nctu.edu.tw/ (accessed on 27 October 2024), WoLF PSORT at http://www.genscript.com/wolf-psort.html (accessed on 27 October 2024), and BUSCA at http://busca.biocomp.unibo.it/ (accessed on 27 October 2024).

### 4.3. Chromosomal Locations and Collinearity Analysis

A genomic annotation dataset was utilized to gather the chromosomal positioning data for 83 *RcGST* genes. The chromosomal distribution of these *RcGST* genes was visualized using TBtools https://github.com/CJ-Chen/TBtools/releases [42], displaying only those genes that corresponded to specific chromosomes. The analysis of collinearity among the *RcGST* genes within the rose genome was conducted employing the Multiple Collinearity Scan toolkit (MCScanX, http://www.megasoftware.net/, accessed on 27 October 2024) with the software’s preset settings. Collinearity was also examined using the standard parameters provided by MCScanX [43]. Genes situated within a 100 kb range on the same chromosome were classified as part of a tandem duplication event, whereas those positioned outside this range were identified as segmental duplications. The syntenic relationships were graphically represented in the form of a circular plot, again utilizing TBtools [42].

### 4.4. Gene Structure and Protein Motif Analysis

Details regarding the exon–intron architecture of the *RcGSTs* were derived from the genomic annotation data of the rose species [1]. The sequence and classification of the conserved motifs were investigated using the MEME online tool, Version 5.4.1, available at http://meme-suite.org/tools/meme (accessed on 28 October 2024). The analysis parameters were configured with the following settings: motif distribution set to any number of repetitions; the upper limit for the number of motifs was set to 10; and the motif length was allowed to range from 6 to 200 amino acids. The outcomes, which included the gene structure and motif arrangement, were organized in accordance with the phylogenetic relationships and visualized employing TBtools [42].

### 4.5. Functional Analysis

Gene Ontology (GO) annotation for rose proteins was conducted through BLASTP searches against the Swiss-Prot/UniProt database using a stringent E-value cutoff of 1 × 10^−5^. KEGG pathway annotation was performed using the KofamKOALA web server (https://www.genome.jp/tools/kofamkoala/, accessed on 28 October 2024) with default parameters. Significant enrichment of GO terms and KEGG pathways (FDR-adjusted *p*-value ≤ 0.05) was identified through TBtools [42], which also generated corresponding visualization outputs.

To investigate potential functional associations among RcGST members, we constructed a protein interaction network for the 83 identified RcGST proteins using STRING v11.5 (https://string-db.org/, accessed on 30 October 2024) [44], with subsequent network visualization and topological analysis conducted in Cytoscape 3.6.1 [45].

### 4.6. RNA-Seq Data Analysis

For the comprehensive analysis of the *RcGST* gene family expression profiles in roses, we obtained two RNA-seq datasets from the NCBI Sequence Read Archive (SRA) database. The first dataset, with the BioProject accession number PRJNA546486, encompassed transcriptome data from various tissues of the ‘Old Blush’ rose, including the root, stem, leaf, prickle, stamen, pistil, and ovary [29]. The second dataset, with the BioProject accession number PRJNA351281, contained transcriptome data from different developmental stages of ‘Old Blush’ rose petals, specifically green petals in the flower bud (FB_GP), color-changing petals in the flower bud (FB_CP), pink petals in the flower bud (FB_PP), and pink petals in the open flower (OF_PP) [30]. Both datasets were accessed on 18 January 2024.

The integrity of the raw sequencing reads was assessed utilizing FastQC, version 0.11.8, available at https://www.bioinformatics.babraham.ac.uk/projects/fastqc/, (accessed on 30 October 2024). Subsequent to the removal of adapter sequences with Trimmomatic [46], the purified reads were aligned to the rose genome using HISAT2 with the software’s preset parameters [47]. The expression levels of transcripts for each sample were determined by StringTie’s quantification tool [48]. The transcript expression was quantified using the transcripts per kilobase million (TPM) method, which accounts for the gene transcript levels. For visualization in heatmaps, the TPM values were converted to a logarithmic scale as log2(TPM + 1). The thresholds for differential expression were set at |log_2_(fold change)| ≥ 1 and adjusted *p*-value < 0.05, and the analysis was performed using the DESeq2 R package http://www.bioconductor.org/packages/release/bioc/html/DESeq2.html (accessed on 12 March 2025) [49]. The heatmaps depicting gene expression were generated with TBtools [42].

### 4.7. Plant Material

Plants of rose (*Rosa chinensis*, ‘Old Blush’) and tobacco (*Nicotiana benthamiana*) were cultivated in a greenhouse facility at Shanxi Agricultural University, JinZhong, China, under controlled environmental conditions with a 12 h photoperiod at 25 °C and a 12 h scotoperiod at 18 °C. Sample collection was performed on well-established one-year-old rosa plants, with samples being promptly stored in 5 mL centrifuge tubes post-harvest. Petals were collected at four distinct developmental phases, designated as follows: FB_GP for green petals within the bud, FB_CP for petals undergoing color transition within the bud, FB_PP for pink-hued petals within the bud, and OF_PP for pink petals on fully opened flowers. In addition to petals, other plant parts including roots, stems, leaves, prickles, stamens, pistils, and ovaries were also harvested from the ‘Old Blush’ rose plants for photographic documentation.

### 4.8. Measurement of Anthocyanin Content

The quantification of total anthocyanins was conducted based on the method described by Ubi et al. [50]. For the extraction, around 0.5 g of sample was placed in 5 mL of the extraction medium, which consisted of 1% hydrochloric acid in methanol, and allowed to stand for 24 h at ambient temperature in a protected-from-light environment. Following this, the mixture was centrifuged at 15,000× *g* r/min for 15 min to separate the supernatant. The supernatant’s absorbance was then recorded at wavelengths of 530 nm. The anthocyanin content was quantified as nmol of cyanidin-3-galactoside per gram of sample, based on a molar extinction coefficient of 3.43 × 10^4^ (Ubi et al., 2006 [50]). The mean values were calculated from three independent replicates.

### 4.9. Quantitative Real-Time PCR (qRT-PCR) Analysis

Gene expression analysis was conducted through triplicate qRT-PCR runs (ABI 7300) using CTAB-extracted RNA and Transgen Biotech’s cDNA synthesis kit. Reaction mixtures (20 μL) contained 1 μL cDNA, 0.5 μM primers (Table S5), ChamQ SYBR mix (Vazyme), and DEPC water. Cycling parameters: 95 °C/5 min→40 cycles (95 °C/5 s→60 °C/35 s). Normalized expression levels (2^−ΔΔCT^ [51]) used RcActin/MdActin references for rose/apple, respectively.

### 4.10. Molecular Docking

Cyanidin-3,5-diglucoside (Cy3G5G) is the main anthocyanin in ‘Old Blush’ rose [30]. The molecular docking study between the RcGSTF2 protein and Cy3G5G was performed using AutoDock Vina software, version 1.2.x [52]. The three-dimensional structure of RcGSTF2 was predicted using AlphaFold2, version v2.2.4 [53]. The chemical structure of Cy3G5G was obtained from the PubChem database. Following this, the predicted RcGSTF2 structure was preprocessed with AutoDockTools, which involved adding hydrogen atoms, removing any crystallographic water molecules, and eliminating unnecessary ligands. The Cy3G5G ligand was converted from SDF format to PDBQT format, and hydrogen atoms were added to it using MGLTools, version 1.5.7 [54]. For the molecular docking process, a docking box was defined to encompass the potential active site of RcGSTF2, and AutoDock Vina was employed to predict the binding modes of Cy3G5G with RcGSTF2. The binding mode with the highest score from the docking results was selected and converted from PDBQT to PDB format using MGLTools for further analysis. Finally, the selected binding mode was visualized using PyMOL software, version 3.1.1 [55], allowing us to adjust the display style to observe the interactions between the protein and ligand and to mark key residues and hydrogen bonds, among other significant interactions.

### 4.11. Genetic Transformation of RcGSTF2 in Rose Plants

The coding sequence of *RcGSTF2* was cloned into the pNC-Cam2304-MCS35S vector to generate the overexpression construct (*RcGSTF2*-OE), with the empty vector (EV-OE) serving as a control [56]. Primers used for cloning are listed in Table S5. Both constructs were transformed into *Agrobacterium tumefaciens* strain EHA105. For transient overexpression, ‘Old Blush’ rose leaves were selected due to technical challenges in achieving efficient transformation in petals (e.g., tissue fragility and low infiltration efficiency). Transformed *Agrobacterium* cultures (OD600 = 0.8–1.0) were vacuum-infiltrated into leaves using a protocol adapted from Zhang et al. [57]. Briefly, leaves were infiltrated with EHA105 carrying recombinant vectors and vacuum-treated for 5 min. Following infiltration, the leaves were incubated in the dark at 24 °C for 24 h to promote T-DNA transfer. Subsequently, leaves were maintained under controlled conditions: 21 °C, 16 h photoperiod (light intensity: 150 µmol·m^−^^2^·s^−^^1^), and 60% relative humidity. At 4 days post-infection, the leaves were harvested for phenotypic observation, RNA extraction, and anthocyanin quantification.

For gene silencing, the coding sequence of *RcGSTF2* was cloned into the TRV vector to generate the silencing construct (*RcGSTF2*-TRV), with the empty TRV vector (EV-TRV) as a control. Recombinant TRV vectors were transformed into *Agrobacterium tumefaciens* strain EHA105. Petals were selected for VIGS due to their phenotypic relevance to anthocyanin accumulation and floral coloration. Transformed Agrobacterium cultures (OD600 = 1.0–1.2) were injected into the receptacle of flower buds at the FB_CP stage (color-changing petals in buds), following the method of Liu et al. [58]. Injections were performed using a sterile syringe (1 mL volume), and plants were maintained under standard greenhouse conditions for to observe anthocyanin-deficient phenotypes.

### 4.12. Subcellular Localization Analysis of RcGSTF2

To determine the cellular compartmentalization of *RcGSTF2*, we generated a C-terminal GFP fusion construct by cloning the coding sequence (excluding the stop codon) into the pNC-Cam1304-GFP vector using nimble cloning [56]. The verified 35S:*RcGSTF2*-GFP plasmid was transformed into Agrobacterium tumefaciens GV3101 and selected on kanamycin-containing media. Positive colonies were confirmed by PCR amplification and Sanger sequencing and then cultured in YEB medium supplemented with antibiotics until reaching an OD_600_ of 1.0–1.5. For transient expression, bacterial suspensions (resuspended in infiltration buffer) were syringe-infiltrated into the abaxial epidermis of Nicotiana benthamiana leaves. GFP fluorescence signals were captured 60 h post-infiltration using a Leica TCS-SP8 confocal laser scanning microscope (Leica Microsystems, Germany), with empty GFP vector infiltrations serving as localization controls.

### 4.13. Data Analysis

All statistical analyses were implemented in SPSS 26.0, with significant differences (*p* < 0.05) determined by one-way ANOVA and Duncan’s post-hoc testing. Quantitative results were visualized through Excel 2016-generated graphics.

## 5. Conclusions

In this study, we identified 83 GST genes in the ‘Old Blush’ cultivar of *Rosa chinensis* and characterized *RcGSTF2* as a key regulator of anthocyanin accumulation. The phylogenetic and molecular docking analyses revealed its conserved role in anthocyanin transport, while subcellular localization confirmed its association with the cell membrane. Overexpression of *RcGSTF2* significantly enhanced anthocyanin content in ‘Old Blush’ rose leaves and upregulated biosynthetic genes, whereas silencing in ‘Old Blush’ rose petals led to reduced pigmentation and gene suppression. These results highlight *RcGSTF2*’s dual function in transport and transcriptional regulation, providing a molecular basis for improving rose coloration through genetic engineering. Our findings advance the understanding of the GST-mediated anthocyanin metabolism and offer practical strategies for breeding roses with enhanced ornamental value.

## Data Availability

Data are contained within the article and Supplementary Materials.

## Supplementary Materials

The following supporting information can be downloaded at https://www.mdpi.com/article/10.3390/plants14060932/s1. Table S1: The gene or protein features of GST members in rose; Table S2: Amino acid sequences of 83 *RcGST* genes; Table S3: Detailed motif sequence information of RcGSTs; Table S4: Transcriptome sequencing (RNA-seq) data of *RcGST* genes in different tissues; Table S5: Primers used in this study; Figure S1. Heterologous overexpression of *RcGSTF2* enhances anthocyanin accumulation. Overexpression of *RcGSTF2* in apple peels (A) and calli (D); Anthocyanin content increase in *RcGSTF2*-overexpressing apple peels (B) and calli (E); *RcGSTF2* expression in transgenic apple peels (C) and calli (F). The *MdACTIN* gene was used as the internal control. In panels B, C, E, and F, error bars represent the standard deviation (SD) from three independent experiments, each comprising three technical replicates. Data labeled with different lowercase letters indicate significant differences at *p* < 0.05.
