# Supplementary material for: Identification of the GST Gene Family and Functional Analysis of RcGSTF2 Related to Anthocyanin in Rosa chinensis ‘Old Blush’
Source: Plants (Basel). 2025 Mar 16;14(6):932. doi: 10.3390/plants14060932 (PMC11944598; doi:10.3390/plants14060932)
Supplement: Supplementary file 1 [file plants-14-00932-s001.zip › plants-3523912-supplementary.pdf]

Table S1: The gene or protein features of GST members in rose.

Subfamily	Gene name	Locus Name	cDNA (bp)	CDS (bp)	PP (aa)	MW (kDa)	pI	Subcellular localization	strand
DHAR	*RcDHAR1*	Chr1g0365431	2720	642	214	23.71	6.31	Cy[a,b,c], Pm[a]	+
	RcDHAR2	Chr3g0493051	5360	789	263	29.35	9.18	Mt[a], Cp[a,b,c]	+
EF1Bγ	*RcEF1Bγ1*	Chr7g0220411	3385	1266	422	47.71	6.02	Cy[a,b,c], Cp[b]	-
	RcEF1Bγ2	Chr7g0229531	3425	810	270	30.26	5.19	Cy[a,b,c]	-
	RcEF1Bγ3	Chr7g0229581	2403	393	131	14.77	10.38	Nu[a,c], Cy[b]	-
	RcEF1Bγ4	Chr7g0229711	1455	684	228	25.43	6.24	Pm[a], Cy[a,b,c]	-
	RcEF1Bγ5	Chr7g0230101	3673	750	250	27.75	9.15	Cp[a,b,c]	-
	RcEF1Bγ6	Chr7g0230191	3652	1257	419	47.66	5.7	Cy[a,b,c], Cp[b]	-
GHR	*RcGHR1*	Chr5g0020071	2785	987	329	37.47	6.72	Mt[a], Nu[a,b,c], Cy[b]	-
Phi	*RcGSTF1*	Chr2g0152311	2950	645	215	24.62	5.47	Cy[a], Cp[b], Mt[b]	-
	RcGSTF2	Chr3g0488551	2319	645	215	24.45	7.71	Cy[a,b], Mt[b], Ec[c]	-
	RcGSTF3	Chr6g0293301	3182	657	219	24.7	5.27	Cy[a,c], Cp[b], Mt[b]	+
	RcGSTF4	Chr6g0293321	1456	648	216	23.98	5.89	Cy[a,c], Cp[b]	+
	RcGSTF5	Chr6g0293331	972	360	120	13.51	8.48	Ec[a], Mt[a], Cy[b,c]	+
	RcGSTF6	Chr6g0293341	1742	645	215	23.88	6.09	Cy[a], Cp[a,b,c]	+
	RcGSTF7	Chr6g0303771	1313	657	219	24.83	6.01	Cy[a], Cp[b], Nu[c]	+
	RcGSTF8	Chr7g0215041	1367	654	218	24.47	6.6	Cy[a], Cp[b], Ec[c]	-
Lambda	*RcGSTL1*	Chr1g0364661	2821	891	297	33.35	8.75	Cp[a,c], Cy[b]	-
	RcGSTL2	Chr3g0468151	2337	711	237	27.15	5.84	Cy[a,b,c]	+
	RcGSTL3	Chr3g0468161	2342	711	237	27.18	5.57	Cy[a,b,c], Nu[a,b]	+

Theta	*RcGSTT1*	Chr7g0198031	3058	828	276	31.5	9.55	Cy[a,c], Mt[a], Cp[b]	+
Tau	*RcGSTU1*	Chr1g0327741	521	363	121	13.68	7.9	Cy[a,b], Cp[c]	-
	RcGSTU2	Chr1g0339611	1154	693	231	26.71	6.04	Cy[a,b], Nu[c]	-
	RcGSTU3	Chr1g0339631	1007	693	231	26.77	5.86	Cy[a,b,c]	-
	RcGSTU4	Chr1g0339651	3909	693	231	26.62	5.86	Cy[a,b], Nu[c]	-
	RcGSTU5	Chr1g0339671	809	375	125	14.31	5.2	Cy[a,b], Nu[c]	-
	RcGSTU6	Chr1g0339691	297	297	99	11.15	9.56	Ec[a], Mt[a], Cp[a,b]	-
	RcGSTU7	Chr1g0339761	689	522	174	19.77	5.79	Cy[a,b,c]	-
	RcGSTU8	Chr1g0339771	839	672	224	25.91	5.75	Cy[a,c], Cp[b],	-
	RcGSTU9	Chr1g0339781	1064	693	231	26.58	6.04	Cy[a,b,c], Cp[b],	-
	RcGSTU10	Chr1g0364471	1209	558	186	21.44	6.12	Cy[a,c], Cp[b]	+
	RcGSTU11	Chr1g0364481	758	666	222	25.74	5.47	Cy[a,b,c]	+
	RcGSTU12	Chr1g0364501	867	681	227	26.17	6.25	Cy[a,c], Cp[b]	+
	RcGSTU13	Chr3g0464521	942	654	218	25.22	5.93	Cy[a,b], Nu[c]	-
	RcGSTU14	Chr3g0464531	1086	681	227	25.87	5.78	Cy[a,b,c]	-
	RcGSTU15	Chr3g0464541	838	669	223	25.6	7.62	Cy[a,b], Nu[c]	-
	RcGSTU16	Chr3g0475891	983	621	207	23.93	8.47	Cy[a,c], Cp[b]	+
	RcGSTU17	Chr4g0396351	1847	684	228	26.35	5.25	Cy[a,b,c]	-
	RcGSTU18	Chr4g0396391	537	363	121	14.2	5.72	Cy[a,b,c], Ec[a]	-
	RcGSTU19	Chr4g0396421	960	678	226	25.67	6.54	Cy[a,b,c]	-
	RcGSTU20	Chr4g0396431	2367	702	234	26.94	6.19	Cy[a,b,c]	-
	RcGSTU21	Chr4g0396461	1216	642	214	24.59	5.91	Cy[a,b,c]	-
	RcGSTU22	Chr4g0396521	702	468	156	18.11	5.82	Cy[a,c], Cp[b]	-
	RcGSTU23	Chr4g0400161	683	384	128	14.6	7.82	Cy[a,b,c], Nu[b]	+

RcGSTU24	Chr4g0445201	2085	702	234	25.71	6.85	$Cy^{a,c}$, Cp^{b}	+
RcGSTU25	Chr5g0026581	1711	660	220	25.47	6.85	$Cy^{a,b,c}$	+
RcGSTU26	Chr5g0027291	1655	663	221	25.46	5.62	$Cy^{a,b}$, Nu^{c}	+
RcGSTU27	Chr5g0027311	1343	657	219	25.33	7.64	$Cy^{a,b}$, Ec^{c}	+
RcGSTU28	Chr5g0027331	1775	660	220	25.51	8.25	$Cy^{a,b,c}$	+
RcGSTU29	Chr5g0027341	1989	657	219	25.12	5.62	$Cy^{a,b,c}$	-
RcGSTU30	Chr5g0027351	1475	660	220	25.1	5.38	$Cy^{a,c}$, Nu^{b}	-
RcGSTU31	Chr5g0027361	1146	765	255	29.25	5.33	$Cy^{a,c}$, Nu^{b}	-
RcGSTU32	Chr5g0027381	764	651	217	24.65	5.68	$Cy^{a,c}$, Cp^{b}	-
RcGSTU33	Chr5g0027401	5568	654	218	24.53	5.24	$Cy^{a,b,c}$	-
RcGSTU34	Chr5g0060741	666	453	151	16.7	5.93	$Cy^{a,c}$ $Cp^{a,b}$	+
RcGSTU35	Chr5g0067311	977	663	221	24.71	5.54	$Cy^{a,b,c}$	+
RcGSTU36	Chr6g0252711	511	372	124	13.97	6.2	$Cy^{a,b,c}$	+
RcGSTU37	Chr6g0285721	943	627	209	24.12	5.3	$Cy^{a,b}$, Cp^{c}	-
RcGSTU38	Chr6g0285731	992	675	225	25.44	5.03	$Cy^{a,b,c}$	+
RcGSTU39	Chr6g0285741	1034	678	226	25.95	5.7	$Cy^{a,c}$, Cp^{b}	+
RcGSTU40	Chr6g0285761	1898	693	231	26.97	6.41	$Cy^{a,b,c}$	+
RcGSTU41	Chr6g0285771	872	672	224	25.65	5.58	$Cy^{a,c}$, Cp^{b}	+
RcGSTU42	Chr6g0285781	829	669	223	26.02	6.26	$Cy^{a,b,c}$	+
RcGSTU43	Chr6g0285791	2051	408	136	15.7	6.32	$Cy^{a,c}$, Mt^{b}	+
RcGSTU44	Chr6g0285821	14164	672	224	25.62	6.14	$Cy^{a,c}$, Cp^{b}	+
RcGSTU45	Chr6g0285831	937	714	238	27.92	8.71	$Cy^{a,c}$, Mt^{b}	+
RcGSTU46	Chr7g0179081	808	651	217	25.02	5.71	$Cy^{a,c}$, Cp^{b}	-
RcGSTU47	Chr7g0179091	1159	642	214	24.44	5.49	Cy^{a}, Cp^{b}	-

	RcGSTU48	Chr7g0179101	827	678	226	26.04	5.63	Cy[a,b], Nu[c]	-
	RcGSTU49	Chr7g0181061	1925	699	233	26.2	5.83	Cy[a,b,c]	-
	RcGSTU50	Chr7g0181081	1561	765	255	28.87	6.52	Cy[a,b,c]	-
	RcGSTU51	Chr7g0181091	1066	699	233	26.11	6.02	Cy[a,b,c]	-
	RcGSTU52	Chr7g0181111	1079	705	235	26.88	7.88	Cy[a,b], Cp[c]	-
	RcGSTU53	Chr7g0181251	1120	696	232	26.32	5.56	Cy[a,b,c]	-
	RcGSTU54	Chr7g0181301	637	552	184	21.32	6.1	Cy[a], Mt[b], Nu[c]	+
	RcGSTU55	Chr7g0181321	1169	702	234	26.97	6.01	Cy[a,b], Nu[c]	+
	RcGSTU56	Chr7g0215201	1530	675	225	25.88	6.02	Cy[a,c], Cp[b]	+
	RcGSTU57	Chr7g0237741	1470	699	233	26.27	5.74	Cy[a,b,c]	+
Zeta	*RcGSTZ1*	Chr1g0350551	5387	819	273	30.96	8.63	Mt[a], Cp[b,c]	-
	RcGSTZ2	Chr7g0218201	4601	657	219	24.86	6.44	Cy[a], Mt[a], Cp[b,c]	+
	RcGSTZ3	Chr7g0218231	4386	657	219	24.62	5.96	Cy[a], Cp[b], Nu[c]	+
	RcGSTZ4	Chr7g0218251	4698	669	223	24.99	4.94	Cy[a,b], Nu[c]	+
TCHQD	*RcTCHQD1*	Chr6g0296751	2542	804	268	31.63	9.25	Mt[a], Ec[b], Cp[c]	-

Table S2: Amino acid sequences of 83 *RcGST* genes

>RcEF1Bγ2

MLVNVSVHYVLHAGNTNKNGYKLIAAEFSGVNVEMVKDFEMGLSNKTPEFLKMNPIGKVGVVSFAGVGSCVPVLETPDGPVFESNAIARYVTH
LKADNPLYGSSLIEYALVEQWIDFATLEIDANILRWFIPRIGFAVYLPPASGTCMIQRVTLFGFAITNTMMHEKTVSFVTLNKVGGFLQRMDLARR
YAFGKMLVIGSDPPFKGKGLWLFRGQEVPQFIIDECYDMELYDWHKVDITDEDQKEHVNQMIEVQEPFEGEALLDAKCFK

>RcEF1Bγ3

MPIFCVGSYRFSVLQSTFAEEAAISSLKRALTALNTHLASNTYLVGHSVTLADIIMICNLTLGFKRLLTKNFTSEFPHVERYFWKLVNQPNFRKILG
EVKQAESVPAVQSAKKPSQPSLRNLQRKRPRKK

>RcGSTU16

MADEVILLDAYASVFGMRVRVALAEKGIKYEYREEDLRNKSQRLLKMNPVRKKIPVFIHNGKPVCESLVIVHYIDEVWKDKAPLLPSDPYQRAQ
ARFWADFIDKKLYDAIRKIWPAKGEEQEAGKKEFIEVLKIWVPAFLDIALITFYSCFHACETLGNFSIEAECPKLISWAKRCLQKESVSKSLADPKK
VYENLLEMEKRLGVE

>RcDHAR2

MSTTTVRIHPTASALSSTIKHHLLVRPSHRHVVFRNNNSFSRRGMVSMAATPLPVCVKPSVTVPNKLGDCPFCQRVLLTLEEKHLPYELKLVDLA
HKPEWFLKINPQGKVPVVKLDDKWIADSDIITQILEDKYPEPPLTTPPEKASVGSKIFSTFIGFLKSKDPKDGTEQALISELSTFNDYLKDNGPFVNG
EKVSSVDLSLAPKLYHMEIALGHYKSWSVPDSLPYVKSYLKRIFSLDSFTKTSALKEDVIVGWRSKVLG

>RcGSTL1

MTSTLKVSFRGVVPTSSPLKSRPRVLDSLYVAKFPNTTILCPPKLRLQAKTTRASLSATMATGVQEALPPALTSTSDPPSIFDGKTRLYISYTCPFAQ
RAWIARNCKGLEEKIELVPINLQDRPSWYKEKVYPPNKVPSLEHNNEVKGESLDLIRYIDSNFEGPSLFPDDPAKREFAEELFTYTDSFSKPVISFFK
GEGTEAAAGAAFDYIETALAKFEDGPFFLGQFSLVDIAYAPFIDRFQHFALDVKKYDITAGRPKLAAWIEEMNKNEGFNKTRRDPKELVESYKKR
FSAKV

>RcGSTU55

MEGESKVKLHGMWASTYSKRVELALKLKGIPYEYIEEDLRNKSQLLLKYNPVHKKIPVLVHNGKPIAESYIILEYIDETWKNAPKLFPEDPYERAR
IRFWASFIQQQLFESMSRIVTSHGEAQEQAVKDAFEKLRVFEEGMKEYLPGGASFANGENLGLLDILMVTTFGPHKAHEEVFGIKLMDPERYPLLF
SWITALNEHPLMKEIQPPYDKLVELLQHFKPTNQATGTESH

>RcGSTF1

MVVKVYGPSYASPKRVLVCLVEKEVEFETIPIDLFKREHKDPEFLKLQPFGAVPVIQDGDYTLYESRAIIRYYAEKYKSQGTDLLGKTIEERGLVE
QWLEVEAQNYHPSIVNLVIHILFAPAQGLPSDSKIIQESEEKLGKVLDVYEERLSKNKYLAGDFFSLADLSHLPFTQYLVGAIGKDYMIKSREHVSA
WWDDISNRPSWKKVLEFGAPF
>RcGSTF4
MAGIKVHGTPFSTAAARVLATLYEKEVEFEFVPIDMRAGEHKKESFLALNPFGQVPAFEDGDLQLFESRAITQYIAHEYAPKGTPLIFPDSKKMAIL
SVWTEVEAQKYDPVASKLTFELVIKPLMLGLATDFTVVEEFEAKLGTVLDVYEARLGKSKYLGGDCFSLADLHHLPTTDYLMGTQVKKLFECRP
NVSAWVADITARPAWKKVVAMRAH
>RcEF1Bγ4
MHALLIPVPVLETPDGPVFESNAIARYVTRLKVDNPLYGSSLIEYVLVEQWIDFTTLEIDANILCWFIPHIGFAVYLPPAEEAAISSLKRALTALNTH
LASNMYLIGHSVTLADIIMICNLTLGFNRLLTKSFTSEFPRVERYFWTLVNQPNFHKILGEVKQAESVPAVQSNKPSQPKEPAKKEAKKEVKKEPA
KPKVEEAAQEEEAPKPKAKNPLDLLPPSKMILD
>RcGSTF3
MVLKLHGLSVSTNTARVVACLHEKDVDFELVRVDLFARGNKEPSFLAKNPFGQIPVLEDGDITLFESRAITSYVAEKFKETGTDLIRHESFDEAAL
VKVWTEVESQQYHPAICPIIYEFFGKPVQGLEPDQAVIDANLEKLEKVLNVYEAKLSSTKYLAGDFYSLADLHHFPYTFYFMKTAWGSAISDRPH
VKAWWDDITSRPALKKVAEGMTFGENQ
>RcGSTU17
MEEVKLLGFWPSNFVYRVIWALKLKGVEYDYIEEDVLSNKSELLLQYNPVYKKVPVLVHRGKPIVESCVILEYIEETWPENPLLPEDAHDKAVAR
FWMQFASPDRIPSFTAFFLLPTAGEEREKTIKDVLETLRTLEEQGLGDKKFFGGDSIGLVDLVHGWLASWFEISQEMVGVKLLEPNTLPRLHAWV
QNFKDTPVIKDNLPDYNELLAHMTRVRDRKCLSLRSL
>RcGSTU57
MSGERVKLLGNWASPSALRVKWTLKLKEIEYEYVEEDLPNKSPLLLKYNPVHKKIPVLVHGGKPIAESLVILEYLDETWKQNPILPEDPYERAQA
RFWAKFVDEKCAPGIMSAFTKKGEEKEKAAKEARENLKILESGLGEKQFFGGESIGFVDIAAGWIGIWARLVEEIAEVNLIDTVNLIDTETMPVLD
TWFKRVLEVPIIKECLPPQDKLLEHNKGFHKMLTGGSRGSS
>RcEF1Bγ5

MVSNKTPQFLKMNPIGKVGVMSFAGPVLETPDGPVFESNAIARYVTRLKADNPLYGSSLIEYALVEQWIDFATLEIDANILRWFIPRVGFAVYLPP
AEEAAISSLKRALTALNTHLASNTYLVGHSVTLADIIMILGEVKQAESVPAVQSKKPSQPKEPAKKEAKKEVKKEPAKPKVEEAAQEEEAPRPKA
KNPLDLLPPSKMILDEWKRLYSNTKTNFREVAVKGDSLLTLFIHAFKQILFLIYHKPL
>RcGSTU53
MVEENVVSLHGMWASPFSKSVELALKTEGIPFEFVDEDLRNKSPSLLKYNLVHKKVPVFVHNGKPIAESLIILEYIDETWKTGPKLLQEDPYKRAQ
VRFWASFLQQHVFETMSLLCKTDGEVQEKAIKELFEKLKTFEEGIKDLFPDGAPSIDCSNNLGLLDIVLCSQFGPHKVQEEVLGITTIDPEKNPLLFT
WLKSLNELPLVKELTPHEKLVSVLHFFRNYALKSSAA
>RcGSTU19
MEEVKLLGFWPSSFVYRVIWALKLKGVKFDYIEEDLSNKSELLLQYNPVRKNVPVLVHGGKSIAESLVILEYIEETWPQNPLLPKDPHEKALARF
WMQFGVDKIRPAIFAYFRAIGEEGKKAKEFQEVLKIVEEQGLGNQNFFGGDNTIGLVDIAFGMLAYWLECIEEVVGVKVLEASSLPRLHAWAQN
FKQVPVVKDNLPDRGKLLAHYKHSREKLILAASSSQ
>RcGSTU18
MEEVKLLGFWPSPFVYRVIWALKLKGVEYDYIEEDVLSNKSELLLQYNPVYKKVPVLVHRGKPIVESCVILEYIEETWPENPLLPEDAHDKAVAR
FWMQFASRKLDIIWKRKMICGLNEA
>RcGSTU54
MRKFITHKYLFLSISYFDCAYSKRVELALRLKSIPYEYMEEDLSNKSQLLLEYNPVHKKVPVLIHNGKPISESHIILEYIEETWKTAPKLLPEDPYER
AKVRFWGSFIQQQLFESILKLVTRHGEAQEKAATEVIERQGVFEEGMKKYFGGGASFTNEKNWGLLDILMGSTFGAYEVHEETSN
>RcGHR1
MARSALDEVSQSGAFVRSASEFRNFISRDPNSQFPAEAGRYHLYISYACPWASRCLGYLNIKGLQKAISFTSVKPIWERTKESDEHMGWVFPASDK
ELAGAEPDPLNGAKSIRELYEIASTKYTGKYTVPVLWDKKLGTIVSNESSEIIRMFNTEFNSIAENPALDLYPDHLQSQVNQTNEWIYDKINNGVY
KCGFAKKQEPYDEAVKQLYEALDRCEEILSKQRYLCGNTLSEADIRLFVTLIRFDEVYAVHFKCNKKLLREYPNLFNYTKDIFQVPGMSSTVQMD
HIKRHYYGSHPSINPFGIVPSGPNIDYSSPHGRNKFSASLL
>RcGSTU12
MAQVTLLGAWPSPFVYRVIWALKLKGVDYEYVEEDILYNKSERLLKYNPVHKMVPVLVHAGKPIAESTVILEYIEETWPQNPLLPKDPHGRALA
RFWTKFGEDKNPALFGFLRTEGEQQVRATKEGQEQLRILEEQGLRDKKFFGGDEIGMADLEFGWLALWLEVWSEITGVTLIEAESFPRLHAWIQR
FKEFPTIKETLPDRSAMLTHYKGYKGLRTTYIALAKS

>RcGSTU10
MAQVTLLGFWPSSFVYKVIWALKLKGLDYEYVEEDVLYNKSGRLLKYNPVHKKNPLLPKDPHERALALFWTKFGEDKIPALCGFFITEGEQQVK
ATKEEHEQLRILEEQGLGDKKFFGGDEIGMADLAFRSLAWWLPVMSEIAGVKVIEAESFPRLHAWTQRFKEFPTIKETLPDRSALLTYFKD
>RcGSTU11
MEEVKVIGFWSSPYVYRVTWALNLKGVEYEYQEEDVFNKSDLLLQYNPVHKKVPVFVHGGKRIAESTVILEYIEEAWPQNPLLPTDPHARAMAR
FWTKFGDDKPDFFGFFKTAGEEQIKAAKEAQECLKIIEEHGLEDKKFFDGDKIGMTDLSLGWLAFWLEAMEGAAGVQVLEVNSFPRLHAWIRNF
KEVPVIKENHPDQTRLLAYFKWLREMYTKPATT
>RcGSTF2
MVVKVYGPVRAACPQRVMVCLLEKGVEFEVVHVDLQAGEQKQPHILARQPFGQVPAIEDGNFKLFESRAIVRYYAAKYADRGPNLLGTTLEEK
ALVDQWLEVESHNFNDLVYAVVLQLVVLPSMGETSDLALVRACEEKLKKVFDVYEERLSKSTYLAGRSFTLADLSHLPAIRFLMDEVKMGHLV
TERKNVTAWWEKISNRPAWKKLMRLAQY
>RcGSTU35
MSKAEVILLDCWISPFCMRVKIALEEKGVAYESQAEDLFGGKSELLLTSNPHGKVPVLLHNGKPVSESAIIVAYIDESWSSSPLLPPCAYGRAQARF
WADYIDKKVFDAGKAIFMSKGEAVEVGKKDFIEIIKTLEKALGDKDFFNGDTFGFVDIIGIAMTSWFPAYEKFGSFKLEDHCPKFSAWIKRSWQRE
SVAKVIPEAEKVIEFVTMFRKMMGAED
>RcGSTT1
MNPIRRGWGCKKVRSFSRAENEEEEKMKLKVYVDRMSQPSRAILIFCKVNGIEFEEVQINLAKQQQKSPDFKKINPMGQVPTIADGRFNLFESHAI
LVYLACAFPGVADHWYPADLFRRAKINSVLDWHHFNLRRGAMTYVLNTVLAPVFGLRLNPQAAAEAEKLLSSSLSKIESIWLKGNGKFLLGGFQ
PSIADLSLVCEIMQLELLDENDRSRILGPHKKVLEWIENTKNATRPHFEEVHQILYRAKTRFQEQRSMGGNNTNLSKRLGLPSKM
>RcEF1Bγ1
MALVLHAGSTNKNAYKTLIAAEYTGVKVALAPNFEMGVSNKTPEFLKMNPIGKVPVLETPDGPIFESNAIARYVTRLKADNPLYGSSSIDYAHIE
QWIDFGSLEIDANIVNWFRPRMGKTVYLPPAEEAAIAALKRALGALNTHLASCTYLVGDSVTLADIIMTCNLFMGFTKLMTKSFTSEFPHVERYF
WTMVNQPNFKKVLGEVKQTDSVPAVQSAKKPAQAKESAKPKAKEEPKKEAKKEQAKPKAEAEEEEAPKPKPKNPLDLLPPSKMILDDWKRLYS
NTKTNFREVAIKGFWDMYDPEGYSLWFCDYKYNDENTVSFVTLNKVGGFLQRMDLARKYAFGKMLIIGADPPYKVKGLWLFRGPEIPEFVMNE
CYDMELFEWTKVDISDENQKERVSQMIEDQEPFEGEPLLDAKCFK
>RcGSTU34

MAKNDVKLLGAWPSTFVLRVRIALNVKSVEYEFLQETMEPKSELLLKSNPVHKKIPVLLHNDKPISESLIIVEYIDEAWASGPSILPSGPYERAVAR
FWAAYIDEKWFPAMKGIAAALVLLEDAFQKISKGKSFFGGDHIGYLDVALGGE
>RcGSTU36
MVKLFCFEDSLGTEAQRCPLLLQHNPIHKKVPVLVHNGKSIAESLVILEYIEETWKQNSLLPQDPHDKAAARFWAKFGDDKIFPPIVDTLCSEEKE
QEEAIVKAKGKLKYLEGVLTLAFAKHN
>RcGSTU56
MAQVTLLGFFHSQFVYRVIWALKLKGVDYEYVEEEGLLYNKSDRLLKYNPVHKKVPVLVHDGKPIAESAVILEYIEETWPQNPLLPKDPHGRAL
ARFWTKFGEDKTLALCGFFITEGEQQVKATKEAQEQLRILEEQGLGDKKFFGGDEIGMVDLEFGWLARWLEVMSETAEVKVIEAESFPRLHAWI
QRFKEIPTIKENLPDRSAMLTYFKGRRATFLALAKS
>RcGSTL2
MAYTQEVLPTPLDATSNPPPLFDGTTRLYTYYSCPFAQRVWITRNYKGLQDQIKLVPINLQNRPAWYKEKVYPENKVPALEHNGKIIGESLDLIKY
VDSNFEGPSLFPKDPEKAKFGEELISHVGTFTGALYTAFKANETVKQADAQFDYLENALKKFDDGPFFLGQFSLADIAYIPFVERFQSFLSEVWKY
DIAAGRPKLAAWLEEINKIDAYKVTKTDPKELVGFYKKRFLEQQ
>RcGSTL3
MAYTQEDLPTPLDATSNPPPLFDGTTRLYTYYSCPFAQRVWITRNYKGLQDQIKLVPLNLQNRPAWYKEKVYPENKVPALEHNGKIIGESLDLIK
YVDSNFEGPSLFPKDPEKAKFGEELISHVGTFTGALYTAFKANDTVKQADAQFDYLENALKKFDDGPFFLGQFSLADIAYIPFVERFQSFLSEVWK
YDITAGRPKLAAWLEEINKIDAYKVTKTDPKELVGFYKKRFLEQQ
>RcGSTZ1
MKFYHNPSHLHSLVSSIIKSSKRTQLNNFTRCFTTRVGFESPRTSRPMENTEASYSSSSKLVLYSYWQSSCSWRVRFALNLKGLSYEYKPVNLAKG
EQFNPDFERLNPLHYVPVLVDGDTVISDSYAIFLYLEEKYPQRPLLPADPRLKALNLQGASIISSSIQPLHMLSMLKYIEEKIGPEESLSWAQLHIEK
GFCALEKLLKDFASRFATGNEVYMTDVFLAPQIAIATARFNINMSTFPSLSRVNESYKGLPEFQASSPERQPDAVHGQ
>RcGSTU26
MVEEVVVLGFWSSMFAMRPRVALTEKGVEYEYREEDLPNKTSLLLEMNPIHKKVPVLIHNGKPVCESANIVQYIDEAWKDKGPTLLPSDPYQRG
QARFWVDYIDKKLYEAGKNIRSTKGEEQEAAKKVFLEILKLLEGELGDKTYFGGQSFGFLDINLVTFYCWFLTYETIGNFSIEAECPKLIAWFNRC
TQKESVSKTLPDPKKVYEFCLFLQKYYGVA
>RcGSTU28

MADEVILLDGFASIFGMRVRVALAEKAIKYEYREEDLRNKSQLLLKMNPIHKKIPVLIHNGKPVCESLIIVQYIDEVWKDKAPLLPSDPYQRAQAR
FWADFIDKKLYDAGRKIWITKGEELQAAKKEFIESLKVLEGELGDKPYFMGERFGFLDIALITFYTWFHAYETLGNFSMEAECPKLISWAKRCLQ
KESVSKSLADQKKAYEYLLEMKKRLGVE
>RcGSTU46
MGEVKVLGASLSLFCCRIEWALKQKGITYEYIEEDLRNKSTLLLKSNPVHKKIPVLLHGDKPVAESLVILEYIDETWREYPLMPDDPSEKAMVRF
WAKYVDEKCVISAWTASRTKGHEQEKAIESTQESLKLLNKLIEGKTFFGGETMGFLDLVVGSLPNWIKFIEEFVGIKLFDTKELSFLHEWAQRFTE
IPMIKESIPMKEDLLNYFRAQQNAK
>RcGSTU29
MADEVVLLDFWPSPFGMRLRIALAEKGVKYEYKEEDLRNKSPLLLQSNPVHKKIPVLIHNGKAVCESVIALQYIDEVWTNKPLLPSDPYLRSQAK
FWADFVDKKIYDLGRKTWTSKGDDQEAAKKEFIDCVKLLEVELGDKPFFGGETLGFVDVALVPFYSWFSVYEKFGNFSIEPECPKFIAWVKRCLE
KESVSKSVPDQDKVCDFVVEMRKKLGIE
>RcGSTU47
MGGVKLVATTQSFFCTRVQWALKLKGVEYEIIEEDLRNKSPILLKYNPVYKKVPVLVHDDTPIAESLVILEYIDETWKENPLLPQDPCDRAAARF
WAKFADEKVVFGVWTACTTEGEGKEKAIEAALESLAHLEKQIEGKKFFGGEQIGYLDLVLGWIPHWLNTMEEVGGMKLLEAEKFPCLLEWGQN
SKLHPYSTNQRMHSTQREAYRILDR
>RcGSTU27
MADEVILLDGYVSSFGMRVRVALAEKGIRYEYREEDLRNKSQLLLKMNPIHKKIPVLIHNGKPVCESLIIVQYIDEVWKDKAPLLPSDPYQRAQA
RFWADFIDKKLYDASRKIWTTKGEEQEAAKKEFIEVLKVLEGELGDKPYFMGERFGFLDIALITFYSWFHAFETLGNFSIEAECPKLISWAKRCLQ
KESVSKSLADQKKVYEFVVEMKKSLGV
>RcGSTU48
MGEVKLLGTTQSFPCARIQWALRLKGVEYEFIEEDLRNKSPLLLKYNPVHKKVPVLVHHDKPIAESLVILEYIDETWKENPLLPRDPYDRAMARF
WAKFVDEKVVIAVWGAFTAEGEEKEKAIESALEPLANLEQQIEGKKFFGGEQIGYLDVVVGWISQWLNVMEEVGGMKLLEAERFPFLHEWGQN
FIQAPAIKECIPPREKLVEYFHFSLSYCRRSLAAHKP
>RcGSTU25

MADEVILLDGYVSMFGMRVRVALAEKGIKYEYREEDMRNKSPLLLKMNPIHKKIPVLIHNGKPVCESLIIVQYIDEVWKDKAPLLPSDPYQRAQA
RFWADFIDKKLYDAGKKIWTTKGEEQEAANKEFIEVLKVLEGELGDKPYFMGERFGFLDIALITFYSWFHAYETLGNFSIEAECPKLISWAKRCLQ
KESVSKSLADQKKVYEFVVEMKKRLGVE
>RcGSTF8
MALKLHGLAFSTCTARAATAIYEKGQQFELVPVNLTAGEHKQPGFLAKNPFGQIPVLEDGDLTLFESRAIATYVAEKYKDSGSDLIRHQNLKEAA
LVKVWSEVESQTFHPVISAIVYQFLIAPLFGQQTDQAVIDANLEKLKNILDVYESRLKSSKYLAGDFFSLADLNHCPYLFYFMRSPWTSVVNERPH
VKAWWEDISSRPSFKKVAEGMTFGQK
>RcGSTU13
MGEEVKLFGVWGSPFSRRVEMALKLKGVEYEYFEEDLQNKSDLLLKYNPVHKKIPVLLHKGKPIAESAVILEYIDETWKGFPLLPKDPYQRATAR
FWARFVEEKCMPALFNCTVAEDRKKAVEEAHETLKLLENELKDKKFFGGEDIGFVDIIADFIGGYWLRDIQEIVGLELLTKEKFPKLCEWSDEFV
NHAAIKECLPPRDKLIAFFRTRFGAKP
>RcTCHQD1
MQLYHHPYSIDSQRVRLALEEKGIDYTSFHLNPVTGKNMNASFFRMNPSATLPVFQNGDHKIYNTIEIIQYVERIASVSTGVENMTFSGREVTEWM
HRIQQWNPKFFTLAHIPDKYFRSVSKFLRRVVMARMEESPDLAGAYHRKLQEVYDTEDKLKNSSVLIQDKEHLIRLLDEVEKQLNETTYLAGEEF
TMADVMLIPVLARLVLLNLEDEYIGSRPNTAKYWIMVQQRPSYKKVIASHFNGWRKYRTLWKTWCSIHIRSILRRF
>RcGSTU31
MADEVVLLDFWPSPFGVRLRIALAEKGIKYEYKEEDLENKSPLLLQSNPVYKQIPVLIHNGKAVCESLIALQYIDEVWNDKAPLLPFDPYLRSQAK
FWADFVDKKIYDLGRKACTTKGKEHEAAQKEFMNCIKSLEVELGDKPFFGGETLGVVDVSLVPFFSWFSVYEKFGNFSIELECPKFNAWIKRCLE
KESVSKSLPDQDKIFEIFVDIRKKLGMDRIMTFCGQVVVCRAKSSNILDQFLTLNEFCSCYSE
>RcGSTU7
MEAHSKKMAEVKLFTTWSTPFALRIVWALKLKDVKSDTIFEDLSNKSPLLLQYNPVHKKVPVLVHNGKSVAESFVILEYIEETWKQNPLLSEDPH
ERAAARFWAKFGDEKVLPSIWEAFNSEGKEQEVGIVKAKENVKYLEEELKGKKLFGGEHIGFADIALGWLAEYENLLA
>RcGSTU30
MADEVVLLDYWLSPYGMRLRIALALKGIEYERKEEELNNKSPLLLQSNPVHKKVPVLIHNGKPICESVVALQYIDEVWKDKAPLFPSDPYLRSQA
KFWADYVDKTISGFARKLWSGAKGEEMEAVKKDLFDCLRLFEGELGDKPFFGGETLGLVDLALLPFASWFSVHDKFGTFSLEAECPKIPPWVKR
CFQMESVSESVPSSDQLYDYVVKMRNSRQE

>RcGSTU33
MADEVVLLGFLPSLFAARLKIALAEKGAEYEYKEEDLRSKSPLLVQMNPVHKKVPVLIHNVKPICESLIALQYIDEVWNDKAPLLPSDPGLGAQA
RFWADFIDKKEMIWAAKEEEHEAAKEFFDCIGVLEGQLGDKAFFGGETLGFVDVAVIPFYSWLSVFEKYGNCSIEAKHPKFTAWTKRCMEKESV
SKSLPDQRRIYDFKVGGILTKLSVDQDL
>RcGSTU9
MEAHSKKMAEVKLFRTWSSPAALRIVWALKLKDVQYDTIFEDLSNKSPLLLQYNPVHKKVPVLVHNGKSVAESLVILEYIEETWKQNPLLSEDP
HERAAARFWAKFNDEKVLPSYWEAFNSEGKEQEEGIVKAKENLKYLEEELKGKKFFGGEHIGFADIALGWLAEIENVFGEVASMKVIAEDEFPLL
SEWKRTFADAPIIKENWPPRDKLVTKYQAFREANLLSKARK
>RcGSTU8
MAEVKLFRTWSSPFALRIVWALKLKDVQYDTIFEDLSNKSPLLLQYNPVHKKVPVLVHNGKSVAESFVILEYIEETWKQNPLLSEDPHERAAARC
WAKFGDEKVLPSIWEAFKSEGKEQEEGIVKAKENLKYLEEELKGKKFFGGEHIGFADIALGWLAEYENVFEEVTSMRVIAEEEFPLLSEWKRTFS
DAPIIKENWPPRDKLVTKFQALREANLLKKVPK
>RcGSTU32
MADEVVLLDFWPSLFGMRLRIALAEKGIKHEYKEEDLKNKSPLLLKSNPVHKKIPVLIHNGKPVSESLIALQYIDETWNDNAPLLPSDPYLRSQAR
FWAEFVDKKVADFGRKVCMTKGEEQEAAKNGLLDCIELLEGELGEKPYFGGETLGFVDVTLVPSYGWLSVYEKFGNFSIEAEHPVFIAWGKRC
MQKKSVSKSLPDQQKLYDFVVQFMEKA
>RcGSTF7
MATSVKVYGPPISTAVSRVLACLHEKEVPYQLIPVNMAKGEHKKPEYLKIQPFGQVPAFEDEGFALFESRSICRYICEKYAKQGNKGLYGENPLA
KASIDQWLEAESHSFSPPSSVLVFQLAFAPRMKLKQDQGAIRQNEEKLKKVLDVYEETLRESRFLAGDDFTLADLSHLPNTHYLVNSSDMGELFT
ERDNVGRWWSEISSRESWQKVVEMQKPA
>RcGSTU15
MAGEDVKLYGTWRSPYSRRVEIALKLKGVQHKYYEEDLTNKSPSLLKYNPVHKKIPVLVHGEKVISESHVILEYIDETWKGYDILPKDPCEKAKA
RFWARFIDEKCLPALKKALASMAEERDNAVEEACELLQLLENELKHKKYFGGDCIGLVDIVANFISHWLKVLQEVVGVELLTIKKFPKLCEWSEN
FVCHHAVKECLPPKDKLLTWFHSHYGSGTTSK
>RcGSTU14

MAEEEAVKLFGTWRSPFSRRVEIALKLKGVDFKYYEEDLTNKSHLLLKYNPVHKKIPVLVHNEKPIVESLVILEYIDETWKEGFPILPKDPCQRSH
ARFWARFLDEKCLPAIEEACLRSVDREKAVEEACELLKLLENELKDNKFFGGETIGLVDIVASVISCWLKAFQQVAGVELLTKEKLPKLCEWSDE
FVSHAVIKGCLPPSDKLLASLRAHFETVASKELCT
>RcGSTU38
MEDVKLFGAWPSPYGYRAIWALELKGVKYEYIEEDLSNKSDLLLQYNPIHKKIPVLVHNGKPISESTVILEYIEETWPGNPLLPSLEDPHGRASARF
WTKFIDDKALPFTMLLMTDGEELEKAAKEVKEILKILEEQGLVEKEFFGGNEIGLADLAMGFIASSFGVIEELAGVKVLNGDEFPGLCNWVKKFK
ENPAIKKNLPDPDQMYVYYKQKREMLIASRAA
>RcGSTU39
MAQVILLGAWPSSFVYRVIWALKLKGVDYEYVEEDVLYNKSDRLLKYNPVHKKVPVLVHAGKPIAESAVILEYIEETWPQNPLLPKDPHGRALA
RFWTKFGEDKSSALFGFFFTEGEQQVKATEERQEQLRILEEQGLGDKKFFGGDEIGMADLEFGWLAWWLEVLSEAAGVKVIEVESFPRLHAWIQ
RFKEIPTIKETLPDRSAMLTYFKDRRATILAKILNTS
>RcGSTU24
MAKNDVKLIGAWPSPFVLRARIALNVKSVEYEFLQETMEPKSELLLKSNPVHKKIPVLLHNDKPICESLIIVEYIDEAWASGPSILPSDPYERAIARF
WAAYTDEKWFPAMKGILGAQNDEARMAAVGQVTEGLVLLEEAFQKISKGKSFFGGDHIGYLDIAFGGFLAWIRVTEGMGGIKLLNETNTPGLL
KWAEKFAADPAVKDVLPETQKLAEFSKIIIAKLRAAAAAKAK
>RcGSTU37
MEEVKLLGTWPSPFCYRVIWALKLKGVKYEDVEEDLINKSDRLLQYNPVHKKVPVLVHGGKPILESTVILEYIEEVWPQNPLLSDDPYERATARF
WTKFGEDKHERARVQGQEVLKIMEEQGLGEKKFFGGDKVGLADLAFGWIAGWLQEMEEAAGVKLLEANKFPRLEDWIKNFREVTVIKDNLPG
HEELLDYFRCLRERLALSSTS
>RcGSTF5
MRGGEHKKESFLALNPFGQVPLFQLLKTEIFSSLLWRNLKLKFGTILDVYETRLGQSKYLGGDCFSLADLHHLPTTHYLMETQAKKLFECCPNVS
AWVADITAIRPAWELTKVVAMGTH
>RcGSTF6
MAGIKLHGNALSTAASRVLAALYEKEVEFELVPIDMIAGEHKKESFLALNPFGQVPAFEDGDLQLFESRAITQYIAREYASKGTPLIFPDSKKMAIL
SVWAEVEAHTFDPPASKLKDELAMKPFLGLATDFAVVEEFEAKLGKILDVYETRLGQSKYLGGDCFSLADLHHLPIIHYLMRTQAKKLFECRPNV
SAWVADITARPAWKKVVAMRAY

>RcGSTU43
MAQVTLLGAWPSPFVYRVIWALKLKGVDYEYIEEDVLYKKSDRLLKYNPVHKKVPVLVHAGKPIAESAVILEYIEETWPQNPLLPKDPHGRALA
RFWTKFGEDKSPALFAFFKTEGEQQVKATEERQEQLRILEE
>RcEF1Bγ6
MALVLRAGNTNKNGYKALIAAEFSGVNVELVKDFEMGVSNKSPEFLKMNPIGKVPVLETPDGPVFESNAIARYVTRLKADNPLYGSSLIEYALVE
QWIDFATLEIDANILRWFIPRIGFAVYLPPAEEAAISSLKRALTALNTHLASNTYLVGHSVTLADIIMICNLTLGFNRLLTKSFTSEFPHVERYFWTL
VNQPNFRKILGEVKQAESVPAIQSKKPSQPKEPAKKEAKKEVKKEPAKPKVEEAAQEEEAPKPKAKNPLDLLPPSKMILDEWKRLYSNTKTNFRE
VAVKGFWDMYDPEGYSLWFCDYKYNDENTVSFVTLNKVGGFLQRMDLARKYAFGKMLVIGSDPPFKVKGLWLFRGQEVPQFIIDECYDMELY
DWHKVDITDEDQKERVNQMIEDQEPFEGEALLDAKCFK
>RcGSTU41
MEIVTLLGAWSSPFVYRVIWALKLKGVDYEYIEEDVLYNKSDRLLKYNPVHKKVQVLVLARKSIAESAVILEYIKETWPQNPLLPKDPHGRALAR
FWTKFGEDKSPALFGFFLTEGEQQVKATEEQQEQLRILEEQGLGDKKFFGGDEIGMADLEFGWLAWWLEVLSEATGVKAIEVESFPRLHAWIQR
FKEIPTIKETLPDRSAMLTYFKDGRATILASAKS
>RcGSTU42
MEEVKVIGFWTSPYVHRVTWALKLKGVEYEYQEEDIWNKSDLLLQYNPVHKKVPVFVHGGKTIVESGIILEYIDETWPQNPLVPTDPHARAMAR
FWIKFVGDKIQNFVGFYKRTGEEQIKAVKEAQEYLKILEDHGPEEKKFFDGDNIGMTDLSMAWLAFWLEAMEEASGVQVLEANSFPRLHAWIRN
FKQVPVIKENHPDHTRLVAYFKRLREKFTKPATT
>RcGSTU40
MSRNERNMEEVKVIGFWTSPYVYRVTWALKLKGVEYEYQEEDIWNKSDLLLQYNPVHKKVPVFVHGGKTIVESGIILEYIDETWPQNPLVPTDP
HARAMARFWIKFVGDKMKNFQGFFFKRAGEEQIKAAKEAQEYLKILEDHGPEEKKFFDGDNIGMTDVSMGWLAFWLEAMEEASGVQVLEANS
FPRLHAWIRNFKQVPVIKENHPDHTRLVAYFKPLREKFTKPATT
>RcGSTU21
MADQVRLFGTWCSPFSTRVIWALKLKGIPYDYIEEDLPYKKSAQLLKYNPVHKKIPVLVHGEKPICESMVIVEYIEETWLQNPLLPSDPYGRAMA
RFWVKFAEDTVREEQEKAKKEVLEMLKTIEEHAGLDKKRFFGGDNIGIVDIAFGQIARWFGVIEEVVGMELLEPHAFPLVHAWTNNFKDVPAIRG
NLPDHDRMVAFFKPFRESLLASS
>RcGSTU3

METHSKKMAEVKLFRTWSSRFALRIVWALKLKDVQYDTIFEDLSNKSPLLLQYNPVHKKVPVLGHNGKSVAESLVILEYIEETWKQNPLLSEDPH
ERAVARFWAKFGDEKVLPSIWEAFNSEGKEQEEGIVKAKENLKYLEEELKGKKFFGGEHIGFADIALGWLAEYENVFEEVASMKVIAEDEFPLLS
EWKRTFADAPIIKENWPPRDKLVTRYQAFRETNLLNKAPK
>RcGSTU20
MEEVKLLGFWPSPFVYRVIWALRLKDVQFDYVEEDLSNKSELLLRYNPVLKKVPVLIHGGKAIAESLVILEYIEETWPQNPLLPKDPHEKALARF
WMQFGLEKMRPIHQAYFPAIGEEREKAAKQYQEVLKILEDQCLGDQRFFGGETIGMVDIVYGMLAYWFECMEEAVGVKVIEPSILPRLHAWVK
NLKQVSVINDNLPHHGKLLAYYKTSREKLVSARSTLPTPSNHDAS
>RcGSTU2
METHSKKMAEVKLFRTWSSVFALRIVWALKLKDVQYDTIFEDLSNKSPLLLQYNPVHKKVPVLVHNGKSVAESLVILEYIEETWKQNPLLSEDP
HERAVARFWAKFGDEKVLPSIWEACNSEGKEQEEGIVKAKENLKYLEEELKGKKFFGGEHIGFADIALGWLAEYKNVFEEVASMKVIAEDEFPL
LSEWKRTFADAPIIKENWPPRDKLVTRYQAFRETNLLNKAPK
>RcGSTU52
MAEETRVILHGSRLSPYVKRVEMALKIKGIPYEFVQEDLKNMSPLLLKYNPVHKKIPVLVHNGKPLAESLVILEYIDETWKTSPQLLPEDPYRRAR
VRFWASFLHQQLFEALVLVIRTNGEAQQKAIKQLYNQLKLFEDGMKDLFPDGIPLSVDNKINVGLLDVLMFSVFDSYEAHEQVLGIKVIDPEKTP
VIFSWIKAINELPVVKELHIPHEKVVASVKLFRKLAMNSSSDT
>RcGSTU4
MEAHSKKMAEVKLFRTWSSPFALRTVWALKLKVVQYDTIFEDLSNKSPLLLQYNPVHKKVPVLVHNGKSVAESFVILEYIEETWKQNPLLSEDP
HERAAARFWAKFGDEKVLPSIWEAFNSEGKEQEEGIVKAKENLKYLEEELKGKKFFGGENIGFADIALGWLAEYENVFEEVASMKVIAEDEFPLL
SEWKRTFADAPIIKENWPPRDKLVTKFLAHREANLLQKVPK
>RcDHAR1
MALEVAAKAAAGAPDILGDCPFTHRVLLTLEEKKVPYKLHLINLADKPQWFTEVNPEGKVPVVKFDDKWVSDSDVLAGILEEKYPEPVLKTPPE
FASVGSKIFGSFVTFLKSKDPSDGSEQALLNELKALDEHLKAHGPYIAGEKVTAADLSLAPKLYHLKVALGHFKKWTVPESLTHYHKYTELLFSR
ESFVKTIAEEKHIIAGWEPKVNPK
>RcGSTU5
MKSIVLPSIWEAFNSEGKEQEEGIVKAKENLKYLEEELKGKKFFGGEHIGFADIALGWLAEYENVFEEVTSMKVIAEDEFPLLSEWKRTFADAPIIK
ENWPPRDKLVTKFQALREANLLKKVPK

>RcGSTU6
MEVVKLFRTWSSPFALRIVWALKLKDVQYDTIFEDLSNKSSLLLQYNPVHKKVPVIVHNGKSVAESFVILEYRDGATTRAVWAVAQGKNWAKN
CQVGV
>RcGSTU1
MSAFTKKGEQKGKAAKEARENLKILESGLGEKQFFGGESIGFVDIAAGWIGIWARMNEEIAERNLIDTETMPLLDAWFRRFLEVPIIKECPPPQDK
LLEHMNGFHKMLTGGRIIIIIRRH
>RcGSTU22
MADQVKLYGVWNSPFSCRVMWALKLKGIPYDYIEEDLRNTKSAQLLKYNPVHKKIPVLVHGEKPICESMVIIEYIEESWPQNPLLPTDPYERAMA
RFWVKFADDKGPAIWRAFKSAGEERENAKKETLEMLRTIEERAGLEKKEFSEETTLVLQT
>RcGSTU44
MAIVTLLGAWPSPFVYRVIWALKLKGVDYEYVEEDVLYNKSDRLLKYNPVHKKVPVLVHAGKPIAESAIILEYIEETWPQNPLLPKDPHGRALAR
FWTKFGEDKSPALFGFFFTEGEQQVKATKEGQEQLRILEEQGLGDKKFFGGDEIGMADLEFGWLAWWLEIMSETAGVKVIDAESFPRLHAWIQR
FKEIPTIRETLPDRSAMLTYFKGRRATFLALAKS
>RcGSTU45
MYIYSRNKKMSRKKTNMEEVKVIGFWASPYVYRVTWALNLKGVKYEYQEEDIFNKSGLLLQYNPVHKKVPVFVHGGKLIAESSVILEYIEETWP
QNPLLPTDPHARAMARFWTKFRDDKPDLYGFFKRAGEEQIKAAKEAQECLKILEEHGLQEKKFFDGDKIGMTDLSMGWLAFWFEAMEEAAGV
QVLEANSFPRLHAWIRNFKEVPVVKENHPDQTRLLAYFKWLREMYNKPATT
>RcGSTU50
MTLREMAEQNKVILYGMWTSPYVKRVELALKVKGIRYEYVDEDLRNKSPLLLEFNPIHKKVPVIVHNGKAIVECLRHGLSDQNHWRSTRESHQR
SSEKVTLLESGLQGLFPDGIPSVDEYSKNVALLDLVILAHFGGYEAQEEVLGLKLIDSEKTPLIPAVKESRNPHEKVVAFLKFFRENALKSGNKSPIL
LLKFNSIHEKVPVLVHNGKTIAESLVIIEYIDDIRTTEPQFLPEDPYKTPNSLMGQLYATGE
>RcGSTU51
MAEQDKVILHGMWASPLTKRVEFALKVKGIPYEYVEEDLSNKSPLLLKFNPVHKKVPVLVHNGKAIAESLVIIEYIDETWKTGPQLLPEDPYKRS
QIRFWAGYMQQVFEAMLLVVKTIGEAQEKAIKEVSEKVKLLETGLKGLFPDGIPSVDEYSKNVTLLDLVIFAHFGGYEAQEEVLGLKFVDSEKTP
LVVSCITALIEIPALKESRIPHEKTVAFLKFLRENALKSATA
>RcGSTU23

MKIFPPIVDALCSEGKEQEEAIVKARENLKYLQEELKGKKFFAGKQIGFADIALRWLAHHENVFEEEAASMKLIAEAEFPLLSQWQKTLSDAPIFK
ENWPPKDKLVTKFQAIHEHELVKKLKKGAPK
>RcGSTZ3
MATASDQQLKLYSCYMSSCAYRVRIALNLKGLKYEYKAVNLLKGEQFSPEFRKLNPIGHVPVLVDGDLVVSDSFAILLYLEEKYPQHPLLPKDLQ
RKAINFQAANIVCSSIQPLQNLAVLNYIEERVSPDAKIELAKVHIEKGFAALEDLLKNYAGRYATGDEVSFADLFLAPQIHAAFKWFNLDMTQFPL
LSRLHEAYNEIPAFVDARPDKQPDAPS
>RcGSTZ2
MATAGDQQLKLYSYYRSSCSFRVRIALNLKGLKYEYRAVNLLKKEQYSPEFRKLNPIGYVPVLVDGEMAVSDSFAILLYLEEKYPQHPLLPKDLQ
RKALNFQAANIVCSSIQPLQNLAVLNYIEERVSPDAKIEWAKVHIEKGFAALEDLLRNYAGRYATGDEVSFADLFLAPQIHAAFTRFNLDMTQFPL
LSRLHEAYNEIPAFVDARPDKQPDAPS
>RcGSTU49
MAEQDKVILYGVWASPYVKRVEFALKVKGIPYEYVEEDLRNKSPLLLKFNPVHKKVPVLVHNGKAIAESLVIIEYIDETWKTGPQLLPEDPYKRS
QIRFWASYMQQVFESLVLVIESSGEAQEKAIKEVSEKVKLLEAGLKGLFPDGIPSVDEFSKNVTLLDLFILAHFGEYEAHEEVLGLKLIDSDKTPLV
ASCITALIEIPAVKKSRIPHEKMVAFLKSYREDALKSATA
>RcGSTZ4
MASGSDENGDQQLKLYSYYMSSCAYRVRIALNLKGLKYEYKAVNLLKGEQFSPEFRKLNPICCVPVLVDGDIAVSDSFAILLYLEEKYPQHPLLP
EDLQRKAINFQAANIVCSSIQPLLNLAVLNYIEERVSPDAKIEWAKVHIGKGFAALEDLLKNYAGRYAIGDEVSFADLFLAPQIDASFSRFNLDMT
QFPLLSRLHEAYDEIPAFIDARPDKQPDATS

Table S3: Detailed motif sequence information of RcGSTs.

MotifID	MotifSeq	Length	Evalue	Sequence Features
MEME-1	VHKKVPVLVHNGKPIAESLVILEYIDETW	29	0	Glutathione S-transferase, N-terminal domain
MEME-2	GFWPSPFVYRVRWALKLKGVEYEYIEED	28	0	Glutathione S-transferase, N-terminal domain
MEME-3	DPYERAQARFWAKFGDEKVLPA	22	0	GST_C_family super family
MEME-4	FGGEEIGLADJALGW	15	0	Not Known
MEME-5	GVKVJEAESFPRLHAWIRNFKEVPVIKE	28	0	GST_C_family super family
MEME-6	NKSPLLLKYNP	11	0	Not Known
MEME-7	TEGEEQEKAIKEAQENLKILE	21	0	GST_C_family super family
MEME-8	FYSWFSAYEKFGNFSIEAECPKLIAWAKRCLQKESVSKSLPDQKKVYEFV	50	3.50E-263	GST_C_family super family
MEME-9	NLPPRDKLVAYFKAFRETLLL	21	8.80E-165	Not Known
MEME-10	DNPLYGSSLIEYALVEQWIDFATLEIDANILRWFIPRIGFAVYLPPAEEA	50	1.90E-111	GST_C_family super family
MEME-11	PQNPLLPK	8	6.80E-102	Not Known
MEME-12	TLGFNRLLTKSFTSEFPHVERYFWTLVNQPNFRKILGEVKQAESVPAVQS	50	9.50E-76	GST_C_family super family
MEME-13	AIEKLKKALDVYETRLASSKY	21	2.00E-59	GST_C_family super family
MEME-14	QRKAJNFQAANIVCSSIQPLQNLAVLNYIEERVSPDAKIEWAKVHIEKGF	50	2.00E-57	GST_C_family super family
MEME-15	ERPNVAAWWEDITKRPAWKKV	21	2.30E-57	GST_C_family super family

Table S4: Transcriptome sequencing (RNA-seq) data of *RcGST* genes in different tissues

GeneID	Root	Stem	Leaf	Prickle	Stamen	Pistil ovary	FB_GP	FB_CP	FB_PP	OF_PP
RcEF1B2	1.63415	1.2229245	0.839718	0.7950985	0.6242935	1.50456	7.9306075	6.42344	6.348205	2.9576725
RcEF1B3	2.52527	2.57477	1.273265	2.18027	2.451775	2.92688	4.4116675	4.342975	7.67352	5.27687
RcGSTU16	13.4416	29.3343	25.74455	42.0506	1.46989	6.951115	5.2390625	8.84869	85.10785	30.0264
RcDHAR2	5.271405	13.55825	11.77075	5.072095	5.532185	14.7851	20.293425	12.61668	10.808025	3.889305
RcGSTL1	38.17145	49.0999	40.8295	34.1556	19.3217	34.6416	246.03	231.226	50.480525	127.43275
RcGSTU55	265.9065	0.428051	0.1667705	0.30395	0.225362	0.3237575	0.43376975	0.237131	0.26291225	0.13371075
RcGSTF1	620.7585	46.60795	24.1201	128.9935	9.95673	25.55625	23.147275	27.09223	26.193525	12.675075
RcGSTF4	499.6285	91.86975	415.5685	28.3793	8.72002	126.2805	23.35725	9.854588	6.47134	9.9977125
RcEF1B4	0.1189685	1.47246	0.298549	1.60913	2.11608	0.853741	1.8129515	1.546833	2.5338425	1.11030725
RcGSTF3	4.78322	27.75885	9.41963	81.1892	3.94884	24.78	13.105	5.4365	4.7127825	1.926305
RcGSTU17	195.1475	18.28475	14.741	17.1963	0.335559	26.0544	2.095905	0.376043	0.4202655	1.7530425
RcGSTU57	5.705375	3.41219	4.0258	5.09752	4.33084	5.23448	8.60518	8.804395	8.7018675	8.46359
RcEF1B5	0.897009	1.232655	1.26287	1.230294	1.31913	1.392395	2.0896125	1.916683	2.38831	1.234315
RcGSTU53	10.661835	0	0	0	0	0.3670725	0.24714325	0	0	0.0245243
RcGSTU19	40.9139	3.009505	0.1862355	0	0.416588	0.96391	0.10224325	0.330866	0.207719275	1.040634
RcGSTU18	82.9811	33.3376	11.129855	13.7097	0	6.101755	1.13319825	0.445465	0.29071175	1.876234
RcGSTU54	271.309	1.1899115	0.907795	0.186748	0	1.996865	1.38519975	1.416095	0.71155675	0.053631
RcGHR1	51.0831	37.27955	23.95535	39.58535	11.91855	40.5422	23.205075	23.256	15.33235	14.4409
RcGSTU12	47.8897	0	0	0	0	0	0	0	0	0
RcGSTU10	0.220871	0.125875	0.208409	0.0974682	0.1546165	0.16408365	0.6844105	0.501839	2.3265625	0.7024295
RcGSTU11	216.068	1.4620045	0	0.5217435	2.028385	0.1885135	0.0264545	0.144425	0.362393825	0.569892

RcGSTF2	0	0.7744235	0.1745185	29.74275	0.857655	1.014369	10.0885625	202.671	310.7035	1732.5675
RcGSTU35	0.45607	1.555465	1.230205	0.6044715	0.1028935	0.38504	0.0268755	0.039612	0.045641175	0.627319
RcGSTT1	31.01485	26.32485	31.5496	27.324	14.67525	26.7422	31.559625	28.42563	29.441075	18.935175
RcEF1B1	249.336	202.4	119.2455	182.543	71.6705	217.3175	280.10275	264.187	194.1735	119.2505
RcGSTU34	0	0.56592	0	0.9932525	0.262522	0.6098765	0.26341425	0.368657	0.058590925	0.065087825
RcGSTU36	2.84067	0.51993	0	3.84854	0.84805	0.872334	0.5753725	0.111957	0.71348525	0.190891
RcGSTU56	24.5053	0.963591	58.7108	0.773362	1.272713	4.39542	0.37838625	0.359182	0.146766225	8.5978375
RcGSTL2	114.33	14.58355	1.990685	21.6536	9.041025	22.80435	54.97315	49.64255	27.716475	5.02781
RcGSTL3	67.02565	111.7655	52.201	322.535	57.91005	96.87205	196.598	209.6313	165.0885	83.355225
RcGSTZ1	9.18355	9.827385	4.824715	9.13151	2.76146	7.71877	5.855685	3.436585	2.9776875	1.4784375
RcGSTU26	2.79223	16.56885	1.629255	20.59215	0.33196	1.1043655	7.15224	1.887023	0.2261525	0.200681975
RcGSTU28	15.39305	40.14515	27.31155	50.22755	27.3977	46.1396	40.603925	32.45553	22.572025	4.8934625
RcGSTU46	1.29027	0.6702065	0.349424	0.2761745	0.111231	0.4725675	0.20415	0.110286	0.024884375	0
RcGSTU29	284.7025	14.38805	157.472	10.57205	2.823415	29.65315	214.226	87.92925	24.9499	117.432
RcGSTU47	270.379	3.13575	28.4439	15.75345	1.5314185	14.14305	1.2059375	0.698002	0.3423805	8.6957525
RcGSTU27	226.1215	113.7765	12.95725	960.235	10.406125	42.4139	12.707875	21.31385	36.798	12.05165
RcGSTU48	0.9241825	0	0	0	0.04880805	0	0.20228805	0.018965	0.1839255	0.046994825
RcGSTU25	22.3438	89.70165	27.2684	156.805	133.32	51.2691	28.494175	72.5764	298.18	106.444
RcGSTF8	0.167949	32.8312	126.0565	14.82135	487.8885	5.92183	1.13798	1.86722	12.369675	3.597375
RcGSTU13	911.2195	13.23441	9.172475	29.7357	4.630645	7.77394	2.89457	1.94728	1.6900025	6.6038
RcTCHQD1	7.68082	6.920245	7.08812	5.1433	1.20078	9.17882	12.441475	16.76613	13.52635	1.1048905
RcGSTU31	70.3349	0	0.050405	0	0	0	0.26131475	0	0	0.10027985
RcGSTU7	1.268233	0	0	0	0	0.228803	0.6468245	1.187782	0.4515055	1.42080075
RcGSTU30	0	0.4625885	0	0	0	0	0	0	0	0
RcGSTU33	1.548555	0.7080485	1.09906	0.8855625	0.1927295	0.9588255	0.46751525	0.384822	0.26246475	0.092467875
RcGSTU9	29.9874	2.94852	4.2655	26.5076	2.26253	6.58045	0.99460725	1.078954	0.42937135	24.66915

RcGSTU8	16.1951	0.5084555	0.3870955	0.261272	0	0.125175	0	0	0.03119775	0
RcGSTU32	21.2321	0.0642935	0	0	0	0.124656	0.05229675	0	0	0.0382967
RcGSTF7	2.94058	40.36085	104.2885	13.8283	3.48372	28.99405	67.050975	141.4773	136.813	3.945245
RcGSTU15	396.9415	0	0.0656675	0	0	0	0	0	0	0
RcGSTU14	1.705465	0	0	0	0.107281	0.170221	2.037595	0.145331	0	0
RcGSTU38	30.03205	1.1640685	14.40515	1.586105	0.115745	1.42715	0.0184236	0.117852	0.045065225	1.464675
RcGSTU39	110.6925	1.219665	21.88075	1.727405	0.1374315	2.284635	0	0.043743	0	2.844885
RcGSTU24	0.2862554	19.79455	158.2895	22.4336	244.794	29.435	14.423525	4.227245	3.7498625	71.763775
RcGSTU37	69.666	0.601042	5.29814	1.0914375	0	0.261138	0.0280545	0.156712	0.071006625	0.7564195
RcGSTF5	0	0	0	0	0	0	0	0	0	0
RcGSTF6	17.6031	335.053	1.727705	268.4425	1.185455	163.763	4.6259725	2.333023	1.01047625	2.6077775
RcGSTU43	78.85895	0	2.353585	0.71991	0.1431735	0	0.052447	0	0	3.890225
RcEF1B6	15.0082	41.37845	31.81275	29.2918	9.374225	56.60595	116.15175	97.47235	53.782925	14.624575
RcGSTU41	1.4348545	0.07259	0.0569295	0.058291	0	0.0567925	0	0	0	0.76118825
RcGSTU42	99.4056	1.6562195	2.419515	3.733615	0.5905655	0.877133	3.73062	3.52525	3.6021175	32.0671
RcGSTU40	56.58985	9.11208	13.07445	8.76002	8.769365	10.64417	17.559475	18.04685	16.86275	25.96405
RcGSTU21	119.7125	3.349085	0	8.41357	0.4278205	1.295405	0.042573775	0.105587	0	0
RcGSTU3	98.41885	36.98255	53.4084	131.908	2.48436	73.1916	22.781575	21.3068	8.3575175	13.77105
RcGSTU20	51.8943	7.337745	35.85695	8.64633	5.517705	20.7901	8.4687875	6.39159	5.9970375	22.73
RcGSTU2	103.36695	53.52145	53.6579	309.3175	7.841025	91.40615	22.914475	27.0793	12.7897	16.3489
RcGSTU52	32.0916	1.223391	0.602387	0.4372285	0	1.450156	1.054519	0.109104	0	0.17281675
RcGSTU4	7.028955	2.39958	2.53041	5.026375	1.92415	3.86561	0.83979275	1.06366	1.186555	1.739745
RcDHAR1	98.1817	270.8165	179.721	59.7365	54.83185	213.043	279.66825	206.7855	221.67225	44.204575
RcGSTU5	28.68135	7.45799	7.57839	7.206025	13.2223	9.324525	10.535025	9.500943	8.635035	4.37241
RcGSTU6	0.905215	0	0	0	7.629225	0	0	0.096189	0	0.1187785
RcGSTU1	12.61495	10.04697	9.04834	5.980305	10.579195	9.575195	16.11865	15.6155	18.4717	13.45075

RcGSTU22	39.9776	0.425206	0	3.604095	1.7527575	0.7401285	0.05623375	0.399641	1.0118245	0.03478075
RcGSTU44	487.39	0.3938045	3.33845	0.831532	0.8159535	0.6337855	0.0585535	0.078878	0.060868225	4.1613125
RcGSTU45	10.27663	0.3552435	0.229735	0.566293	0.556608	0.765214	0.5033015	0.251767	1.5152925	1.7544175
RcGSTU50	0	0	0	0	0	0	0.02778325	0.017394	0.03538345	0
RcGSTU51	78.22105	5.0399	2.57687	3.835775	0.9068225	4.62959	4.2502175	2.145118	3.0757625	1.4399175
RcGSTU23	2.884595	1.762245	0.4409065	1.3476	1.74514	3.034745	1.6756175	1.542268	1.258495	0.62979525
RcGSTZ3	46.8965	15.46765	58.768	13.30175	9.955755	32.9379	13.61085	12.26013	10.0993225	7.7149875
RcGSTZ2	43.3756	38.821	49.89575	37.96825	21.4077	56.3829	56.117725	55.57598	40.68415	34.69295
RcGSTU49	75.24705	75.8843	12.49835	39.5171	9.86359	35.7121	23.5636	13.77713	11.546025	7.363695
RcGSTZ4	0.120521	51.8576	19.9486	115.647	0.10796125	6.773695	0.7906025	0.492088	2.0884475	0.349936

Note: The analysis utilized two RNA-seq datasets from the NCBI Sequence Read Archive (SRA) database, specifically PRJNA546486 and PRJNA351281. The transcript expression was quantified using the transcripts per kilobase million (TPM) method, which accounts for the gene transcript levels. For visualization in heatmaps (Figure 5A), the TPM values were converted to a logarithmic scale as log2(TPM + 1). The thresholds for differential expression were set at $|\log2(\text{fold change})| \geq 1$ and adjusted p-value < 0.05, and the analysis was performed using the DESeq2 R package.

Table S5: Primers used in this study.

Genes	Types of primers	Primer sequences (5'-3')	Primers description
RcACTIN	Forward	CAATGCTCCCGCTATGTATG	Reference gene
	Reverse	AGGTCAAGTCGCAGAATGG	
RcGSTF2-OE	Forward	ATGGTAGTGAAAGTTTATGGTC	For transient expression vector construction
	Reverse	CTAGTATTGAGCAAGCC	
RcGSTF2-GFP	Forward	ATGGTAGTGAAAGTTTATGGTC	For subcellular localization vector construction
	Reverse	GTATTGAGCAAGCCTC	
RcGSTF2	Forward	AAGTTTATGGTCCAGTTAG	For qRT-PCR
	Reverse	GTTCCCAGCAGGTTAG	
RcCHS	Forward	CGAGATATCACAATGGTGACCGT	For qRT-PCR
	Reverse	ACATGCGCTGGAATTTCTCCTTGA	
RcF3H	Forward	TTCAAGAACGCCGATCACCA	For qRT-PCR
	Reverse	TGGCTCCTCCAGAATAGGCT	
RcDFR	Forward	CGTAGGTTCATGGCTCGTCA	For qRT-PCR
	Reverse	TCCACAGCGTCAAGTGAGTC	
RcANS	Forward	AGCTCATGGAACGGGTCAAG	For qRT-PCR
	Reverse	TTGCCCGGAAGCATTGTTTG	
RcUFGT	Forward	GAGCCACAAAGTTGCTAGTTCTAG	For qRT-PCR
	Reverse	CTTTTTGCCGGAACTCGAGAAG	

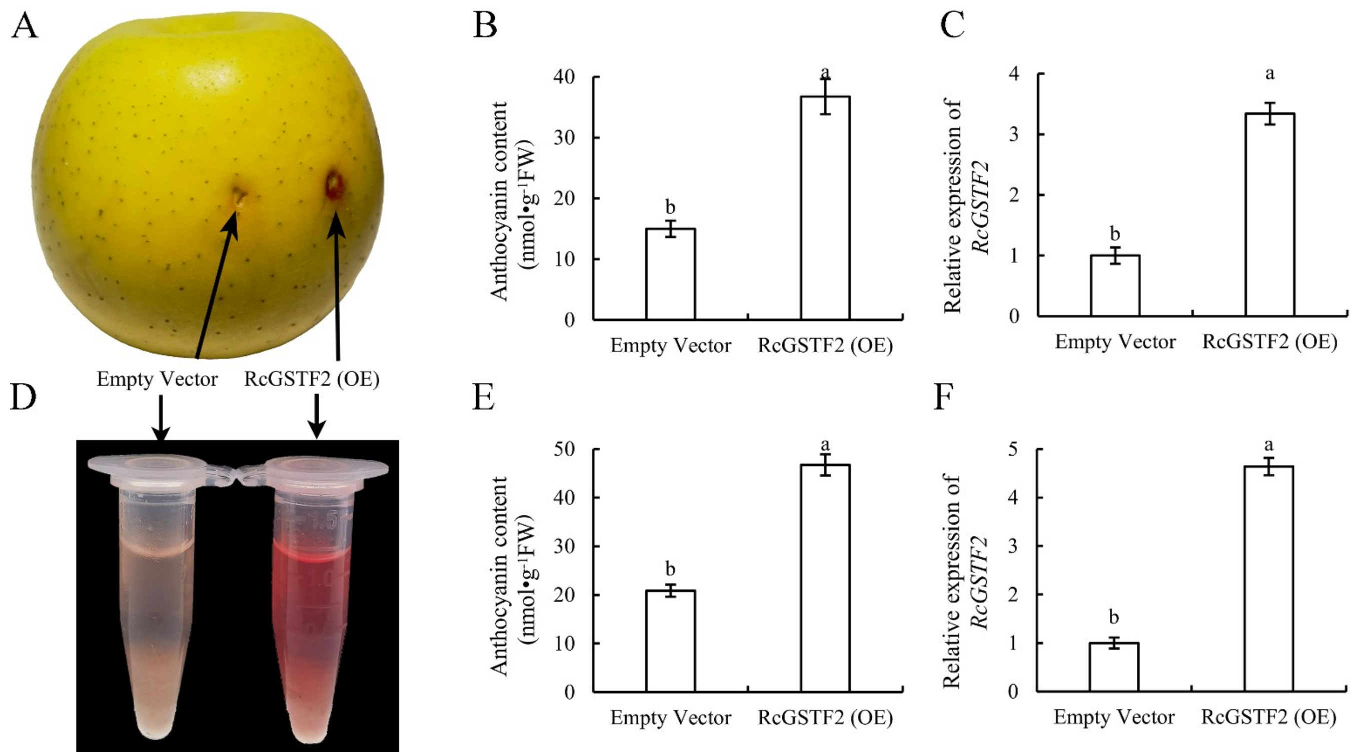

Figure S1. Heterologous overexpression of *RcGSTF2* enhances anthocyanin accumulation. Overexpression of *RcGSTF2* in apple peels (A) and calli (D); Anthocyanin content increase in *RcGSTF2*-overexpressing apple peels (B) and calli (E); *RcGSTF2* expression in transgenic apple peels (C) and calli (F). The *MdACTIN* gene was used as the internal control. In panels B, C, E, and F, error bars represent the standard deviation (SD) from three independent experiments, each comprising three technical replicates. Data labeled with different lowercase letters indicate significant differences at $p < 0.05$.
